# New public QSAR model for carcinogenicity

**DOI:** 10.1186/1752-153X-4-S1-S3

**Published:** 2010-07-29

**Authors:** Natalja Fjodorova, Marjan Vračko, Marjana Novič, Alessandra Roncaglioni, Emilio Benfenati

**Affiliations:** 1 National Institute of Chemistry, Hajdrihova 19, SI-1001 Ljubljana, Slovenia; 2 Institute for Pharmacological Research "Mario Negri", Via La Masa 19, 20156 Milan, Italy

## Abstract

**Background:**

One of the main goals of the new chemical regulation REACH (Registration, Evaluation and Authorization of Chemicals) is to fulfill the gaps in data concerned with properties of chemicals affecting the human health. (Q)SAR models are accepted as a suitable source of information. The EU funded CAESAR project aimed to develop models for prediction of 5 endpoints for regulatory purposes. Carcinogenicity is one of the endpoints under consideration.

**Results:**

Models for prediction of carcinogenic potency according to specific requirements of Chemical regulation were developed. The dataset of 805 non-congeneric chemicals extracted from Carcinogenic Potency Database (CPDBAS) was used. Counter Propagation Artificial Neural Network (CP ANN) algorithm was implemented. In the article two alternative models for prediction carcinogenicity are described. The first model employed eight MDL descriptors (model A) and the second one twelve Dragon descriptors (model B). CAESAR's models have been assessed according to the OECD principles for the validation of QSAR. For the model validity we used a wide series of statistical checks. Models A and B yielded accuracy of training set (644 compounds) equal to 91% and 89% correspondingly; the accuracy of the test set (161 compounds) was 73% and 69%, while the specificity was 69% and 61%, respectively. Sensitivity in both cases was equal to 75%. The accuracy of the leave 20% out cross validation for the training set of models A and B was equal to 66% and 62% respectively. To verify if the models perform correctly on new compounds the external validation was carried out. The external test set was composed of 738 compounds. We obtained accuracy of external validation equal to 61.4% and 60.0%, sensitivity 64.0% and 61.8% and specificity equal to 58.9% and 58.4% respectively for models A and B.

**Conclusion:**

Carcinogenicity is a particularly important endpoint and it is expected that QSAR models will not replace the human experts opinions and conventional methods. However, we believe that combination of several methods will provide useful support to the overall evaluation of carcinogenicity. In present paper models for classification of carcinogenic compounds using MDL and Dragon descriptors were developed. Models could be used to set priorities among chemicals for further testing. The models at the CAESAR site were implemented in java and are publicly accessible.

## Background

Evaluation of chemical toxicity and human health risk of compounds are of primary interest, because it drives much of the current regulatory actions regarding new and existing chemicals. It is estimated that over 30 000 industrial chemicals used in Europe require additional safety testing. Traditional animal testing is very costly and would require the use of extra 10-20 million animal experiments which is contrary to the policy in EU member states to replace, reduce and refine the use of animals in science (the so called 3 Rs policy). In order to support 3 Rs and REACH policies alternative approaches like Quantitative Structure-Activity Relationships (QSARs) were proposed [1].

Between different endpoints carcinogenicity is one of the most essential ones in assessment of human health safety. A lot of models for prediction of carcinogenic potency have been published in recent years [2-6]. Some QSARs models are developed for particular chemical classes (such as amines, nitro compounds, polycyclic aromatic hydrocarbons) [7-9]. A considerable number of expert systems has been created for the prediction of carcinogenicity. In some cases different endpoints such as genotoxicity, mutagenicity and carcinogenicity could be integrated (for review articles see [10-17]). Models for non-congeneric chemicals are of great interest for regulatory use as they involve various classes of chemicals [18,19].

The big challenge in solving the general carcinogenicity prediction problem is to construct a model that would be able to predict carcinogenicity for a wide diversity of molecular structures, spanning an undetermined number of chemical classes and biological mechanisms. Quantitative models based on SMILES [20] for prediction of carcinogenicity were successfully developed [21,22].

Many statistical approaches can be used for prediction of complex endpoint such as carcinogenicity. The CAESAR models are in the area of the data mining models which address complex endpoints. Others models, which have been developed, are based on toxic residues codifying human expert knowledge, such as Oncologic [13], HazardExpert [23], Derek [24], ToxTree [25], or data mining based on fragments, such as MultiCase [10,12]. Within CAESAR, the data mining approach has been improved using a highly verified set of compounds (all chemical structures have been double-checked, and experimental data verified in case of some unusual finding, compared to similar compounds), and adopting a wide series of chemical descriptors. Different algorithms have been developed, this resulted in a series of models and one with better performance has been implemented and reported here.

Predictive power of models is one of the most important characteristics in QSAR modeling. In a recent paper Benigni et al. [26] pointed out that the prediction reliability should be checked by means of an external test set with new chemicals not used in modeling. The state of art and perspectives of predictive models for carcinogenicity are reported in a recent paper [27]. It was stressed that the models for regulatory purposes should be connected with high sensitivity, i.e., the ability to correctly identify true positives. Preliminary results of carcinogenicity modeling using CP ANN algorithm obtained in the scope of CAESAR project are described in an article [28].

Among statistical approaches, artificial neural networks (ANNs) appeared to be one of the most suitable and promising for prediction of complex endpoint such as carcinogenicity for non-congeneric datasets of chemicals. The main advantage of neural network modeling is that the complex, non-linear relationships can be modeled without any assumptions about the form of the model. Large datasets can be examined. Neural networks are able to cope with noisy data and are fault-tolerant [29].

In this paper we presented categorical or qualitative models for prediction of carcinogenic potency of non-congeneric chemicals using CP ANN method. Our models have been developed in accordance with principles of validation adopted by OECD within the European Commission (EC) funded project CAESAR (Computer Assisted Evaluation of industrial chemical Substances According to Regulation) [30].

In our study an external dataset of 738 chemicals was composed and external validation of models made. In the paper it is shown how one can increase the number of correctly predicted carcinogens using correlation between threshold of categorical models and sensitivity and specificity. We address the issue of threshold effects on overall performance of models.

Our final models could serve for the preliminary ranking and prioritization of chemicals for carcinogenic potency, as required by REACH.

## Results and discussion

All CAESAR models for prediction of carcinogenicity were built in accordance with 5 OECD principles [31] and are based on a strict quality assurance/quality control process. In the research activities a parallel or in some cases collaborative work has been done by different partners in the modeling. Indeed, more than one partner worked in the development of models. This allowed a scientific cross validation of the results, because at least indirectly the activity of each group and the results obtained have been discussed and evaluated by all partners during the periodic meeting, in which all models have been presented.

In addition to that, a direct, detailed quality control and double check of the results has been done individually for each of the final model which has been developed and implemented within CAESAR, as present at the web site.

The models at the CAESAR's web site [30] have been implemented in java. This allowed a good portability and a facility of execution within a client-server approach.

### CP ANN models parameters

**Figure 1:**
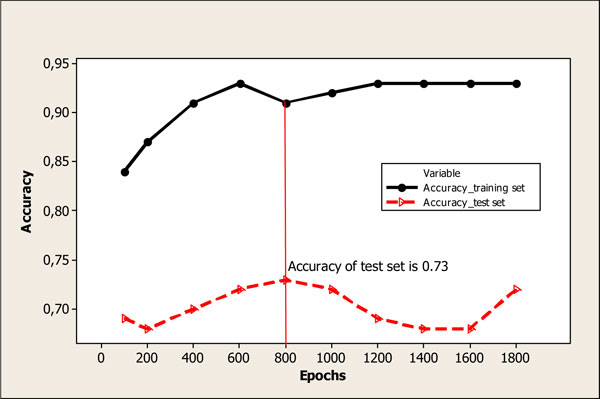
Statistical performance of model with 8 MDL descriptors (model A) and dimension 35*35 depending on number of learning epochs*. *Optimal model corresponds to 800 learning epochs (accuracy of test set is equal to 0.73)

**Figure 2:**
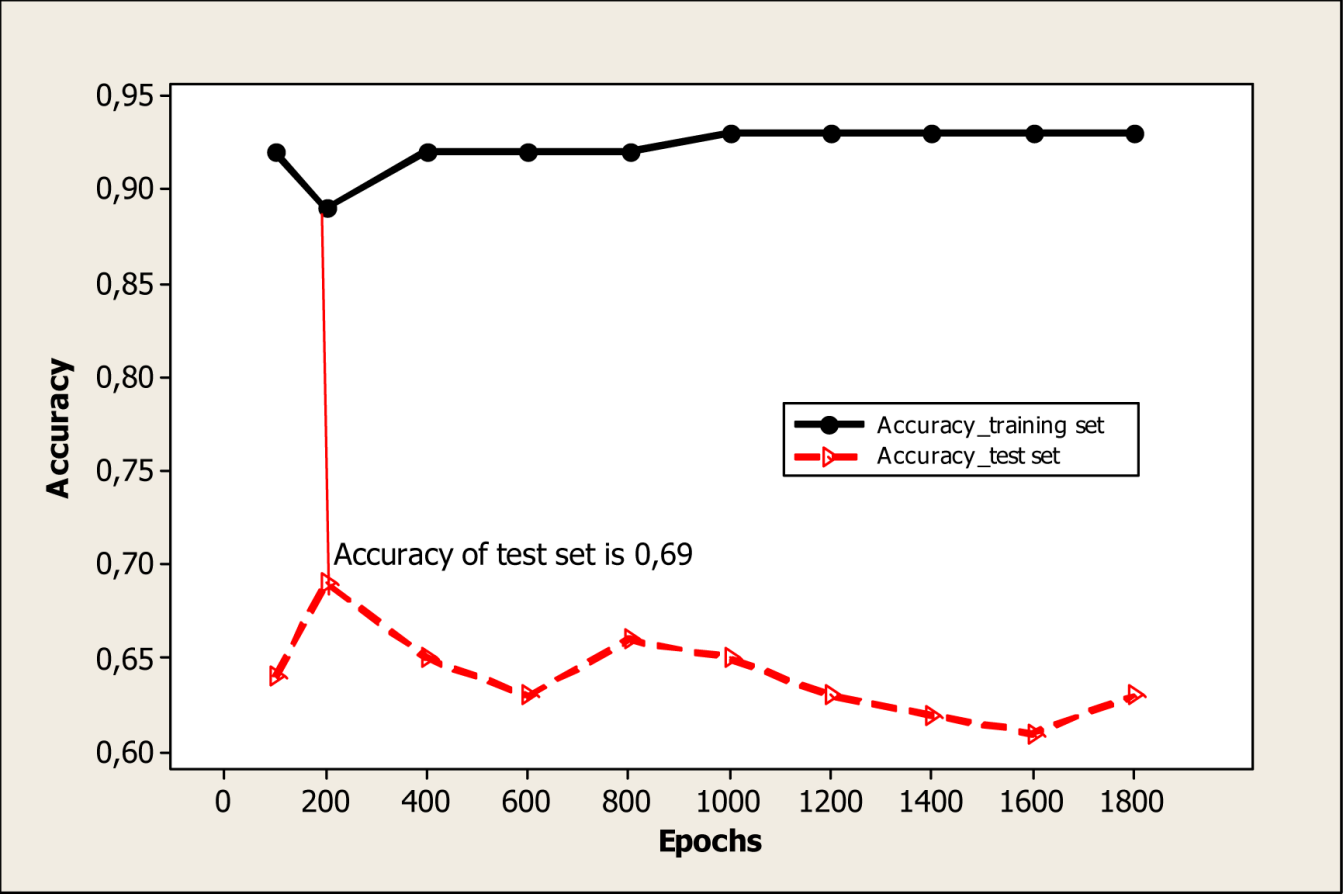
Statistical performance of model with 12 Dragon descriptors (model B) and dimension 35*35 depending on number of learning epochs*. *Optimal model corresponds to 200 learning epochs (accuracy of test set is equal to 0.69)

A considerable amount of models have been built with dimensions 20*20; 25*25; 30*30; 35*35; 40*40 and number of learning epochs from 100 to 1800. Minimal correction factor was set at 0.01. Maximum correction factor was set at 0.5. The highest prediction power was obtained for models with dimension 35*35. Statistical performance of models with dimension 35*35 depending on number of learning epochs is represented on Figure 1 and Figure 2 for models with MDL (model A) and Dragon (model B) descriptors correspondingly. The highest accuracy for test set equal to 0.73 was obtained using 800 learning epochs for model A (see Figure). For model B the highest accuracy for test set was equal to 0.69 which corresponds to 200 learning epochs. Hence, optimal dimension for models with MDL and Dragon descriptors was set equal to 35 *35 neurons. The number of learning epochs was accepted 800 and 200 for optimal models with 8 MDL and 12 Dragon descriptors, correspondingly.

We have used the software developed in the laboratory of chemometrics (National Institute of Chemistry, Ljubljana, Slovenia), written in FORTRAN for IBM-compatible PCs and Windows operating system. This software program "AnnToolbox for Windows" is avalable at home page of National Institute of Chemistry Slovenia [32].

For categorical models a threshold (cut-off) value of 0.45 was applied for models with 8 MDL descriptors and 0.5 for model with 12 Dragon descriptors. Chemicals falling in a terminal node with mean response higher than 0.45 or 0.5 were classified as positive (active or carcinogens) and chemicals falling in a terminal node with mean response lower than 0.45 or 0.5 were classified as negative (inactive or non carcinogens) for model A and B correspondingly.

In all approaches descriptors serve as independent variable and biological activities as dependent.

### Model using eight MDL descriptors

With 8 MDL descriptors and CP ANN algorithms described above we produced dozens of models. After their evaluation one model was accepted as the best one (Model A). The statistical performance of this model is presented in Table 1. The Cooper statistics based on training set indicated an accuracy of 91%, high value of sensitivity (96%) and specificity (86%). As for test set (161 compounds) we obtained accuracy equal to 73%, sensitivity (75%) and specificity (69%). Cross validation (leave 20% out) results gave us accuracy 66%. By results of external validation (738 compounds) we have got accuracy 61.4%. The obtained results indicated that models possessed good stability. Reliability and robustness of model are high as we get good statistical performance for all criteria, on both the internal and external sets.

**Table 1:** Statistical performance of models using 8 MDL descriptors (Model A) and 12 Dragon descriptors (Model B).

	Model A (8 MDL descriptors)		Model B (12 Dragon descriptors)	
**Internal validation**	***Training (644 compounds)***	***Test (161 compounds)***	***Training (644 compounds)***	***Test (161 compounds)***

Accuracy, %	91	73	89	69
Sensitivity, %	96	75	90	75
Specificity, %	86	69	87	61

**Figure 3:**
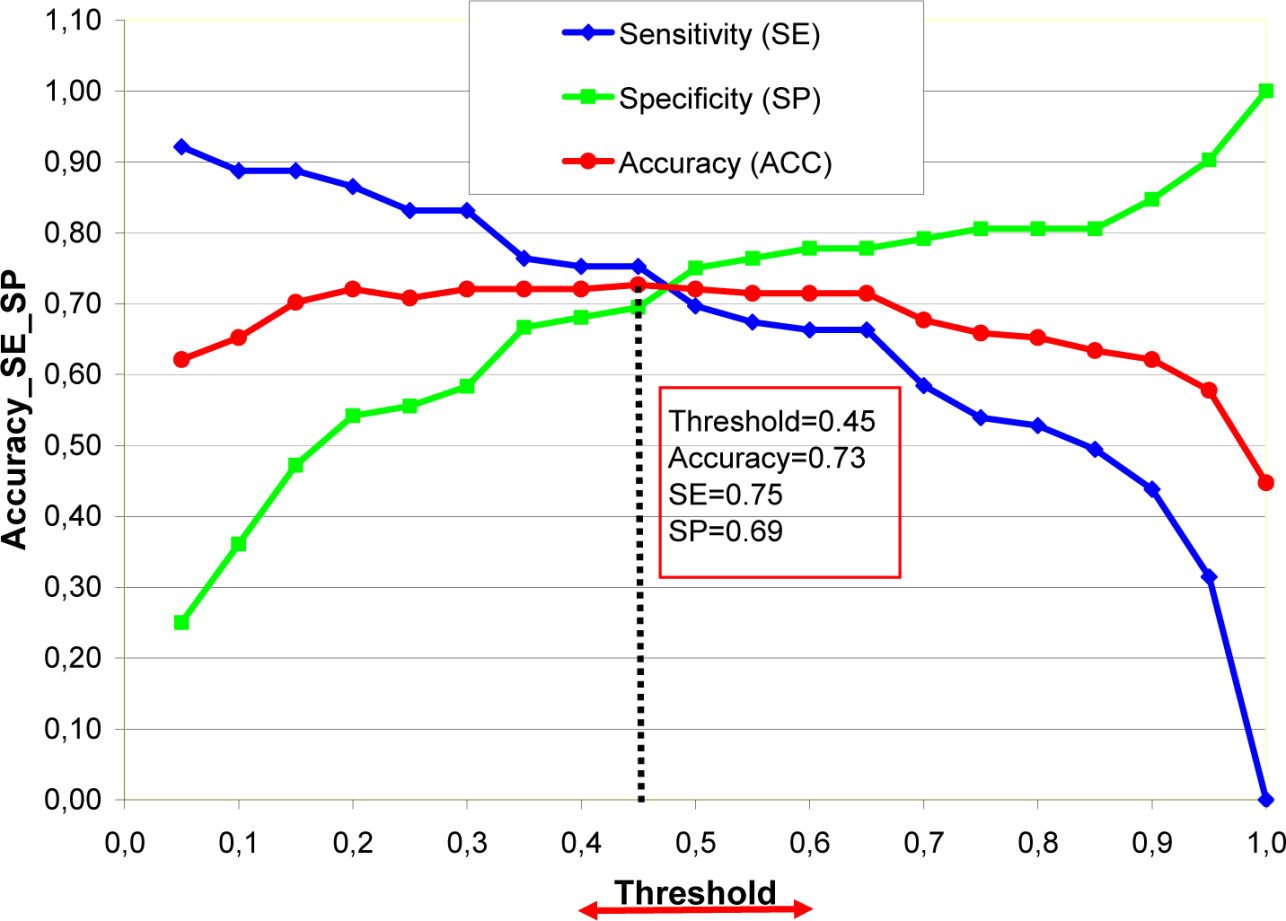
**Accuracy (ACC), sensitivity (SE) and specificity (SP) of test set (161 compounds) vs. threshold for CP ANN model A**.

In the introduction (background) of this article we have pointed that the model reliability should be connected with high sensitivity (correctly predicted carcinogens) to ensure public safety. Therefore in this paper we described how changing threshold of model we can increase sensitivity. Figure 3 shows the accuracy, sensitivity and specificity for test set for model A depending on threshold. From a regulatory perspective, the higher sensitivity in prediction of carcinogens is more desirable than high specificity. Changing the threshold in model A, we can vary sensitivity and specificity depending on needs. In the interval of threshold from 0.05 to 0.9 the accuracy is greater than 60%. Setting on threshold to 0.05 we are able to increase the sensitivity till 90% without considerable reduction of accuracy as it still remain at the level of 60%. From other hand we can keep in mind that increasing of sensitivity leads to considerable reduction of specificity till approximately 20% in case of threshold 0.05 (see Figure 3).

Another important feature of models for regulatory purposes is the reproducibility. Therefore the parameters of model have to be fixed. The user does not need to optimize the model parameters. On the Figure 3 it is shown the optimal model performance corresponding to the threshold equal to 0.45.

As an alternative choice Dragon descriptors can be used for prediction of carcinogenicity using CP ANN algorithm.

### Model using twelve Dragon descriptors

12 Dragon descriptors were selected as was described in section methods. CP ANN algorithm was employed for the dataset of 805 chemicals and used in modeling. We selected an optimal model with dimension of neural network 35*35 and number of learning epochs equal to 200. The threshold was set up at 0.5.

The Cooper statistics of the model with 12 Dragon descriptors based on the training set (644 compounds) indicated the accuracy 89%, sensitivity 90% and specificity 87%, and for the test set (161 compounds) the accuracy was equal to 69%, sensitivity 75% and specificity 61%. The threshold was set at 0.5. The characterization of this model B is given in the Table 1.

### Validation of models using external set of 738 compounds

The CAESAR applet available on the intranet was used to predict a set of 738 compounds which were not used in modelling (not presented in the CAESAR dataset of 805 chemicals). These chemicals were provided with carcinogenicity class assigned on the basis of experiments and extracted from the Leadscope software. The predictions obtained for these compounds are summarized in the misclassification tables (Table 2 and 3 for model with MDL and Dragon descriptors correspondingly).

**Table 2:** Confusion matrix for external validation set of 738 chemicals of the model obtained with MDL descriptors (model A).

		Leadscope experimental carcinogenicity class	
		
		*Carcinogens*	*Non-carcinogens*
**CAESAR predicted carcinogenicity class**	***Carcinogens***	231	155
	
	***Non-carcinogens***	130	222

**Table 3:** Confusion matrix for external validation set of 738 chemicals of the model obtained with Dragon descriptors (model B).

		Leadscope experimental carcinogenicity class	
		
		*Carcinogens*	*Non-carcinogens*
**CAESAR predicted carcinogenicity class**	***Carcinogens***	223	157
	
	***Non-carcinogens***	138	220

Overall the performances obtained with this externally predicted dataset are as follows:

accuracy = 61.4% and 60.0%; sensitivity = 64.0% and 61.8% and specificity = 58.9% and 58.4% respectively for model with MDL and Dragon descriptors.

### Applicability domain of models

The definition of the applicability domain (AD) of a QSAR model is very useful to define boundaries whereby the obtained predicted values can be trusted with confidence. So far no standard solutions have been agreed within the scientific community to optimally define these boundaries, but often the proposed solutions rely on chemometrics methods. The state of art of methods for identifying the domain of applicability of (Q)SARs is given in paper [33]. For carcinogenicity endpoint, as for the models developed for the other endpoints, CAESAR implemented a tool for the general evaluation of the AD based on the descriptor range for the dataset; therefore, predicted values for chemicals outside the descriptor range can be judged as less reliable. The ranges of implemented MDL and Dragon descriptors are presented in Table 4 and 5, correspondingly.

**Table 4:** The range of MDL descriptors for model A

MDL descriptors symbol	Min_value of descriptor	Max_value of descriptor
*SdsCH*	0.000	30.74
*SdssC_acnt*	0.000	18.000
*SdsN_acnt*	0.000	4.000
*dxp9*	-0.4009	7.7061
*nxch6*	0.000	7.000
*Gmin*	-5.0185	2.000
*SHCsats*	0.000	66.2633
*SHBint2_Acnt*	0.000	8.000

**Table 5:** The range of Dragon descriptors for model B

DRAGON descriptors symbol	Min_value of descriptor	Max_value of descriptor
*PW5*	0	0.167
*D/Dr06*	0	1114.64
*MATS2p*	-1.357	1.0
*EEig10x*	-1.0	3.9280
*ESpm11x*	0.693	19. 939
*ESpm09d*	0.0	15.483
*GGI2*	0.0	15.111
*JGI6*	0.0	0.047
*nRNNOx*	0.0	2.0
*nPO4*	0.0	2.0
*N-067*	0.0	2.0
*N-078*	0.0	4.0

This kind of global, chemometric estimation of the AD does not address two key aspects. Firstly, the chemical space characterised by the descriptor range does not take into account the density of compounds distribution, so it might happen that the target chemical falls in an area poorly represented in the training set. Moreover, since the AD relies on the chemical descriptors alone, the output layer (the property under investigation) is neglected. To overcome these aspects, CAESAR developed a further tool for the AD assessment, based on the measurement, through a similarity score, of the six most similar chemicals in the training set; it can be used to evaluate if these compounds are really representative for the unknown compound. Furthermore, a visualisation of these compounds is offered, which can be used to independently evaluate the compounds. Finally, a quantitative report of the error between the observed and predicted activity is also provided for these substances, so that it is possible to argue about wrong behaviour for the model in the chemical area that better represents the compound of interest. The CAESAR models can be accessed through "CAESAR Application" which is a java-based web application [34].

### Structural and chemicals diversity of studies datasets

**Figure 4:**
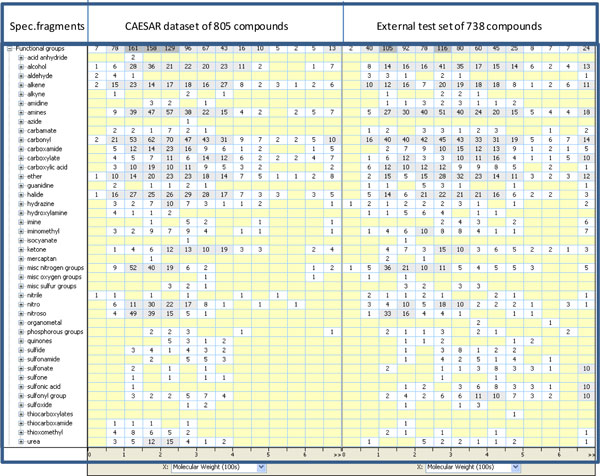
**Representation of CAESAR (left side) and external test set (right panel) in terms of number of chemicals showing specific fragments, as calculated by Leadscope software**.

The structural and chemical diversity of 805 compounds from CAESAR dataset and of 738 chemicals from Leadscope database (external dataset) is illustrated in Figure 4.

In Figure 4 the CAESAR dataset and external dataset of 738 chemicals are represented in terms of number of chemicals presenting some functional groups. It can be noted that the overall picture for CAESAR dataset is quite similar to that of the external dataset. In both data sets the majority of specific fragments are as follows: alcohol, alkene, amines, carbonyl, carboxamide, carboxylate carboxylic acid, ether, halide, hydrazine, ketone, midc nitrogen group, nitro, nitroso, sulfonyl group. In less quantities the following specific fragments are present: aldehyde, alkyne, amidine, carbamate, quanidine, hydroxylamine, imine, iminomethil, isocyanate, mercaptan, misc oxygen group, misc sulfur groups, nitrile, phosphorous groups, quinines, sulfide, sulfonamide, sulfonate, sulfone, sulfonic asid, sulfoxide, thiocarboxamide, thioxomethyl, urea; acid anhydride, azide present only in CAESAR set and 2 fragments: organometal and thiocarboxylates present only in test set.

Additionally we have extracted from Toxtree program specific structural alerts (SAs) for each individual chemical in CAESAR dataset of 805 chemicals. In Table 6 we have listed the number of chemicals with corresponding structural alert. The detailed definition of SAs are published in paper [35].

**Table 6:** The structural diversity of CAESAR dataset of 805 chemicals by presence of specific structural alerts (SAs) extracted from ToxTree program.

Structural Alert (SA)	Number of chemicals
SA_2: alkyl (C<5) or benzyl ester of sulphonic or phosphonic acid	4
SA_3: N-methylol derivatives	2
SA_4: Monohaloalkene	5
SA_5: S or N mustard	7
SA_6 Propiolactones or propiosultones	0
SA_7:Epoxides and aziridines	22
SA_8: Aliphatic halogens	47
SA_9: Alkyl nitrite	1
SA_10: a, b unsaturated carbonyls	0
SA_11: Simple aldehyde	4
SA_12: Quinones	22
SA_13: Hydrazine	32
SA_14: Aliphatic azo and azoxy	7
SA_15: isocyanate and isothiocyanategroups	4
SA_16: alkyl carbamate and thiocarbamate	5
SA_17: Thiocarbonyl	18
SA_18: Polycyclic Aromatic Hydrocarbons	12
SA_19: Heterocyclic Polycyclic Aromatic Hydrocarbons	5
SA_20: (Poly) Halogenated Cycloalkanes	9
SA_21: alkyl and aryl N-nitroso groups	107
SA_22: azide and triazene groups	3
SA_23: aliphatic N-nitro group	1
SA_24: a, b unsaturated aliphatic alkoxy group	1
SA_25: aromatic nitroso group	4
SA_26: aromatic ring N-oxide	1
SA_27: Nitro-aromatic	75
SA_28: primary aromatic amine, hydroxyl amine and its derived esters	52
SA_28bis: Aromatic mono- and dialkylamine	7
SA_28ter: aromatic N-acyl amine	17
SA_29: Aromatic diazo	4
SA_30: Coumarins and Furocoumarins	8
SA_31a: Halogenated benzene	16
SA_31b: Halogenated PAH	7
SA_31c: Halogenated dibenzodioxins	2

### Mechanistic interpretation of model

Development of a structure-information approach which is based on application of different structural descriptors including the electro topological ones shows the new opportunities in prediction of biological activity and properties in contrast to the mechanism based approach [36-38]. The models based on the pointed above approaches are established independent of explicit three-dimensional (3-D) structure information and are directly interpretable in terms of the implicit structure information [39]. The authors [36] demonstrated wide range of applicability of such models for relatively big datasets (e.g. for prediction of aqueous solubility, AMES mutagenicity, fish toxicity and others). In the case of carcinogenicity there are a variety of mechanisms and pathways, including genotoxic and epigenetic ones that might play a role in the observed toxic effect. The application of structure-information approach which is "mechanism-free" makes our task simpler and thus feasible because it is not necessary to assume various mechanistic steps in order to make computations for such complicated biological property like carcinogenicity. This method is free of approximations and computations related to assumed mechanism of interaction. This aspect is very important especially for modelling carcinogenicity using non-congeneric set of substances and aimed for prediction of a wide diversity of chemicals.

The MDL and Dragon chemical descriptors selected within the CAESAR models are presented in Tables 7 and Table 8, correspondingly. The procedure of generation and selection of descriptors is described in section "Methods".

**Table 7:** Eight MDL descriptors selected for modeling.

MDL_ID Descriptor Code	Symbol	Definition
***MDL005***	*SdsCH*	Sum of all (= CH -) E-State values in molecule
***MDL051***	*SdssC_acnt*	Count of all (= C <) groups in molecule
***MDL062***	*SdsN_acnt*	Count of all (= N) groups in molecule
***MDL114***	*dxp9*	Difference simple 9^th ^order path chi indices
***MDL130***	*nxch6*	Number of 6-membered rings
***MDL187***	*Gmin*	Smallest atom E-State value in molecule
***MDL190***	*SHCsats*	Sum of hydrogen E-State on sp3 C on saturated bond
***MDL210***	*SHBint2_Acnt*	Count of internal hydrogen bonds with 2 skeletal bonds between donor and acceptor

**Table 8:** Twelve Dragon descriptors selected for modeling.

Dragon Descriptor's code	Symbol	Definition
***DRA0107***	*PW5*	Path/walk 5 - Randic shape index
***DRA0123***	*D/Dr06*	Distance/detour ring index of order 6
***DRA0341***	*MATS2p*	Moran autocorrelation - lag 2/weighted by atomic polarizabilities
***DRA0391***	*EEig10x*	Eigenvalue 10 from edge adj. matrix weighted by edge degrees
***DRA0451***	*ESpm11x*	Spectral moment 11 from edge adj. matrix weighted by edge degrees
***DRA0464***	*ESpm09d*	Spectral moment 09 from edge adj. matrix weighted by dipole moments
***DRA0551***	*GGI2*	Topological charge index of order 2
***DRA0565***	*JGI6*	Mean topological charge index of order6
***DRA0670***	*nRNNOx*	Number of N-nitroso groups (aliphatic)
***DRA0695***	*nPO4*	Number of phosphates/thiophosphates
***DRA0791***	*N-067*	Al2-NH
***DRA0802***	*N-078*	Ar-N = X/X-N = X

Taking into consideration MDL descriptors (see Table 7), we can see that we deal with electro topological E-state, connectivity and others descriptors. E-state indices are a combination of electronic, topological and valence state information. These indices incorporate information related to atom, types and electron accessibility, hydrogen atom E-states, and connectivities that are influenced by all of the sub-structural features of a molecule [40-42]. Elements identity and skeletal connection contains structure information while valence state definition includes relationship for valence state electro negativity and atom/group molar volume. Based on these important features of molecules, together with skeletal branching pattern, both the electrotopological state (E-state) and molecular connectivity (Chi indices) structure descriptors were successfully implemented for prediction of genotoxicity and carcinogenicity [18,43,44]. The authors [44] contend that one of the critical determining factors for good prediction results depend on nature of molecular structure representation employed in the model development process.

A complete set of *whole molecular descriptors *encode information on general structure features such as molecular size and shape, as well as specific information on skeletal variation and complexity. These structural features are expected to have a relationship to properties arising from intermolecular interactions and may also function to provide discrimination among multiple structural classes.

*The atom-type, group-type, bond-type and single-atom E-state descriptors *encode information on specific molecular features such as atom and bond types associated with important functional groups. Many of descriptors relate directly to or associated with structural alerts as was reported in papers [45,46].

Some of E-state descriptors can be associated with structural alerts for carcinogenicity. For example, in Table 7 the *SdsN_acount *descriptor belongs to atom-type E-State account descriptors and expresses the count for the nitrogen atom type = N-associated with the azo group. The last one is also a structure alert and is correlated with carcinogenicity [44]. In Table 8 nRNNOx and N-078 descriptors are accounting for some specific fragments, whose presence is characterizing for the carcinogenic while the nPO4 descriptor accounts for non carcinogenic class.

*The global E-State descriptor *Gmin is a measure of the most electrophilic atom in the molecule. Mechanistically, an electrophilic center is important for covalent bond formation with nucleophilic DNA. This is the reason why this descriptor was found between the most important descriptors correlated with carcinogenicity.

*Hydrogen E-State descriptor SHCsats *encodes E-state values for hydrogens on sp^3 ^hybrid carbons bonded only other sp^3 ^carbon atoms. The electron accessibility of these sp^3 ^hydrogens may relate in some manner to hydrophobic interactions between substrates and DNA or may have a relation to alkyl chlorides that are known toxicophores.

Thus, the descriptors used in our study refer to topological characteristics as well as to polarizability and charge distribution (related to reactivity).

Interestingly, some descriptors that we applied in our models were also used by others authors [43,44] in carcinogenicity and genotoxicity modelling. It means that probably in future research it will be possible to find some common features for modelling carcinogenicity and genotoxicity. But this issue is not in scope of present study.

It should be highlighted that the application of structure-information approach based on such descriptors like E-State has the following advantage: a model based on E-State descriptors (expressed as continuous value) can correlate carcinogenicity to a specific value of descriptor, whereas the use of fragment based structural alerts limits the model to a correlation of presence or absence of fragments or simple count of given fragments which can lead to false prediction for this reason.

## Conclusion

The CPDB rodent carcinogenic database was used for development of models for categorization of carcinogenic potency. Initial preprocessing of data and selection of data with carcinogenic potency for rats gives us consistent data suitable for QSAR modeling with carcinogenic potency response closer to human. The MDL and Dragon software programs were applied for calculating the molecular descriptors. The topological structure descriptors provided a sound bases for classifying molecular structures.

The CP ANN model presented in our study demonstrated good prediction statistics on the test set of 161 compounds with sensitivity of 75%, specificity of 61%-69% in addition to accuracy 69%-73%. A diverse external validation set of 738 compounds confirmed the robustness of our models regarding a large applicability domain, yielding the accuracy 60.0%-61.4%, sensitivity 61.8%-64.0%, and specificity 58.4%-58.9%.

These carcinogenicity models can be used as a support in risk assessment, for instance, in setting priorities among chemicals for further testing.

The models at the CAESAR's web site have free access for public use [34].

## Experimental

### Internal data set used in modeling

The chemicals involved in the study belong to different chemical classes, so called non-congeneric substances. The work is addressed to industrial chemicals, referring to REACH initiative. The aim is to cover chemical space as much as possible. The initial dataset of 1481 chemicals was taken from Distributed Structure-Searchable Toxicity (DSSTox) Public Database Network http://www.epa.gov/ncct/dsstox/sdf_cpdbas.html which was built from the Lois Gold Carcinogenic Database (CPDB).

The initial dataset has been cleaned of all incorrect structures, ambiguous or mixed structures, polymers, inorganic compounds, metallo-organic compounds, salts, complexes and compounds without well defined structure. The obtained data and structures of chemicals were cross-checked by at least two partner using the following online databases: ChemFinder [47], ChemIDPlus [48] and PubChem Compound [49]. The final data set of 805 chemicals, with their ID number, chemical name, CASRN, experimental TD50 values for rat and corresponding binary carcinogenicity classes are available in Additional file 1 (Table 1SI). For each substance it is indicated whether it belongs to training or test set.

It should be highlighted that data used in our study were obtained from standard protocols and meet requirements for QSAR modeling. Carcinogenicity classification criteria follow the Directive 67/548/EEC, Annex VI, and Cancer Risk Assessment criteria proposed by IARC International Agency for Research of Cancer. Carcinogenicity hazard testing and assessment were performed in accordance with OECD Guidelines 451 (TG 451, 1981-carcinogenicity study).

To prepare data for modeling the dataset of 805 chemicals was subdivided into training (644 chemicals) and test (161 chemicals) sets using the sub-sorting of chemicals according to functional groups and following procedure aimed to distinguish between connectivity aspects. This part of study has been done in the Helmholtz Centre for Environmental Research - UFZ in Germany by a partner in the CAESAR project. The sorting of the compounds pointed here is implemented in the software system ChemProp [50,51].

### External data set used for validation of models

Additional 738 chemicals different from those in our data set of 805 compounds were used as external validation set, being described by the same type of structural descriptors as employed in our model.

To assess predictive abilities of the selected CAESAR model a commercial database has been queried to extract new chemical compounds to be tested. Leadscope software allows accessing some QSAR ready database and the "FDA 2009 SAR Carcinogenicity - SAR Structures" database consisting of 2090 compounds has been extracted from the Leadscope environment in terms of structure information and carcinogenic activity label (based on different mammalian species) and compared with the CAESAR dataset of 805 compounds [52].

The two databases in the form of sdf files as been merged with ChemFinder and specific check to search for duplicates has been performed. The compounds in common between the two sources were analyzed to verify consistency in the experimental carcinogenicity class assigned by the two sources.

A total of 655 compounds were in common and for them the CAESAR assignment was compared with the Leadscope one. The assignment of toxicity class for Leadscope chemicals was based on rat data only and chemicals have been classified as carcinogens if at least one of the two genders (male or female rat) was labelled in Leadscope as positive or intermediate level carcinogen.

Based on this group of 655 compounds the concordance of the two assignments was of 367 positive chemicals and 257 non-carcinogenic ones. Only 31 compounds were classified differently (11 positive in CAESAR dataset but negative for Leadscope and 20 in the opposite situation) hence the overall concordance was above 95%.

Since the concordance between the two experimental sources is very high the Leadscope database can be considered as a reliable source of new compounds to test the CAESAR model.

Once excluded those chemicals already present in the CAESAR dataset it was possible to select as an external test set 738 compounds with experimental data on rats.

Those compounds have been submitted to the CAESAR model to obtain the predicted activity.

### Description of carcinogenic potency

Carcinogenic potency for rats was selected as response because such data in risk assessment [53] are often considered to be more suitable for human carcinogenicity prediction. The term "carcinogen" generally refers to an agent, mixture, or exposure that increases the age-specific incidence of cancer. Carcinogen identification is an activity grounded in the evaluation of the results of scientific research. Tumourgenic dose is accepted for characterization of carcinogenicity. The tumourgenic dose TD_50 _used in our study is defined as the tumourgenic dose rate where 50% of the test animals got any kind of cancer. Using other words, the TD_50 _is that chronic dose rate (in mg/kg body weight/day or mmol/kg body weight/day *(mmol/kg-bw/day)*) which would give half of animal tumors within some standard experiment time, the "standard lifespan" for the species [54]. Chronic oral toxicity and carcinogenicity tests are described in "OECD Environment, Health and Safety Publications Series on Testing and Assessment No 35 Guidance Notes for Analysis and Evaluation of Chronic Toxicity and Carcinogenicity Studies [55].

We have accepted an assignment of carcinogenic categorical activity based on evidence for or against activity within the species group in Target Sites of Rats (Male, Female or Both) as provided in the CPDB Summary Table. Hence, "active" or positive (P) or carcinogen was assigned for a compound if one or more TD50 and the tumor site are listed for one or more rat carcinogenicity sex/species cell (rat male, rat female, rat both) and "inactive" or not positive (NP) or non carcinogens was assigned for a compound if no TD_50 _or tumor site are listed and one or more "no positive results" entry for one or more rat carcinogenicity sex/species cell, i.e. one or more experiments are reported in the CPDB for species, but none are positive. In other words, chemicals were classified as not carcinogenic when the results obtained during all animal tests on rats were assigned as not positive (NP) (or not active) and contrary, compounds were classified as positive (P) (or active) when any of the *in vivo *assays gave a well defined TD_50 _value. In the studied database (805 compounds), 421 chemicals were classified as carcinogenic and remaining 384 as non carcinogens. The distribution of carcinogens and non carcinogens in the total, training and test sets is presented in Table 9.

**Table 9:** The distribution of carcinogens and non-carcinogens in total, training and test sets.

	Total set	Training Set	Test Set
Carcinogens **(P-positive)**	421	332	89
Non-carcinogens **(NP-not positive)**	384	312	72
Totally	**805**	**644**	**161**

## Methods

### Generation and selection of descriptors

Nowadays thousands of chemical descriptors such as constitutional, quantum chemical, topological, geometrical, charge related, semi-empirical, thermodynamic and others can be calculated for chemical structure [56,57].

In present study the following sets of descriptors for 805 compounds have been generated for modeling: 254 MDL descriptors computed using MDL QSAR version 2.2. [58] and 835 Dragon descriptors calculated by Dragon professional 5.4 software [59].

To develop robust and reliable models the descriptors space should be reduced extracting the most significant variables. Variable selection and reduction is a delicate problem. Before the number of descriptors was reduced, all variables were normalized between -1 and +1. In order to select the most relevant descriptors different mathematical tools have been used as listed below.

*Hybrid Selection Algorithm (HSA) *was developed by BioChemics Consulting SAS (BCX), France. This method was used to select the best parameters for classifying the chemicals by their carcinogenic potency (P-carcinogens and NP- non-carcinogens) among the different molecular descriptors series. It combines the Genetic Algorithms (GA) [60,61] concepts and a stepwise regression [62]. In this way the descriptors space was reduced from 254 to 8 MDL descriptors listed in Table 7. Thus, at first, taken into consideration 245 MDL descriptors, we have got the molecular structure information codified as topological descriptors, including atom-type and group-type, E-State and hydrogen E-state indices, molecular connectivity, chi indices, topological polarity, and counts of molecular features [40-42]. Among the eight MDL descriptors there are two connectivity indices (*dxp9*-difference simple 9^th ^order path chi indices and *nxch6*-number of 6-membered rings), three constitutional (*SdssC_acnt- *count of all (= C <) groups in molecule, *SdsN_acnt- *count of all (= N) groups in molecule and *SHBint2_acnt- *count of internal hydrogen bonds with 2 skeletal bonds between donor and acceptor) and three electro-topological parameters (*SdsCH- *sum of all (= CH -) E-State values in molecule, *Gmin- *smallest atom E-State value in molecule, *SHCsats- *sum of hydrogen E-State on sp3 C on saturated bond).

Selection of Dragon descriptors was performed using *cross correlation matrix*,*multicolinearity and fisher ratio techniques *[63]. This part of work was done in cooperation with CAESAR partner (*Central Science Laboratory -CSL Defra, UK*). As a result descriptors space was reduced from 835 to 12 Dragon descriptors listed in Table 8. Among the twelve Dragon descriptors there are 2 topological ones (*PW5***- **path/walk 5 - Randic shape index and *D/Dr06***- **distance/detour ring index of order 6), one 2D autocorrelation index (*MATS2p- *Moran autocorrelation - lag 2/weighted by atomic polarizabilities), two edge adjacency indices (*EEig10x- *eigenvalue 10 from edge adj. matrix weighted by edge degrees and *ESpm11x- *spectral moment 11 from edge adj. matrix weighted by edge degrees), two topological charge indices (*GGI2- *topological charge index of order 2 and *JGI6- *mean topological charge index of order6), two descriptors are from "functional group count" group (*nRNNOx- *number of N-nitroso groups (aliphatic) and *nPO4- *number of phosphates/thiophosphates) and two descriptors are from "atom centered fragments" group (*N-067- *Al2-NH and *N-078- *Ar-N = X/X-N = X).

### CP ANN algorithm

**Figure 5:**
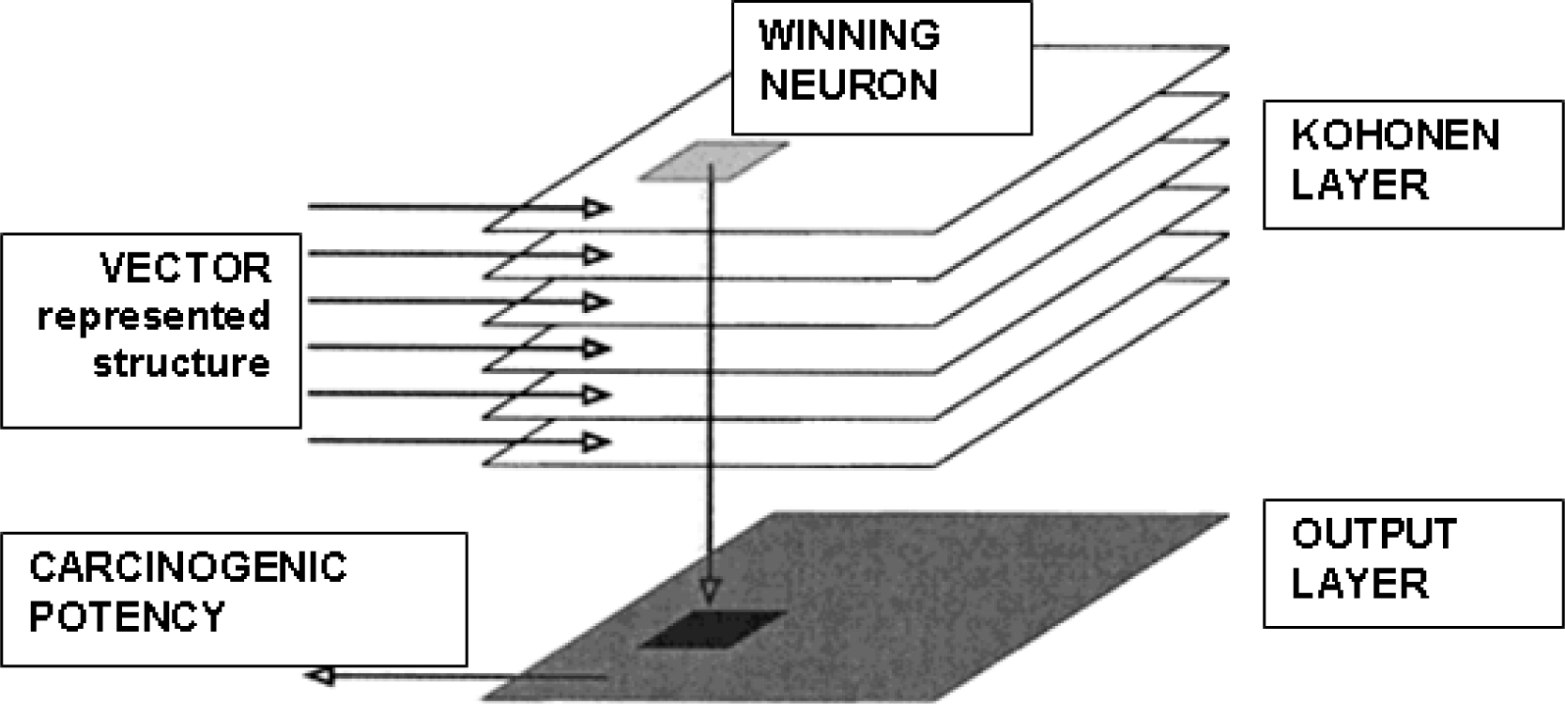
**Counter propagation neural network architecture**.

CP ANN algorithm was used in modeling. In this chapter we give a brief description of the method employed. CP ANN consists of two layers of neurons arranged in a two-dimensional rectangular matrix. The architecture of CP ANN is presented in Figure 5.

In a general way the CPANN can be explained as follows. The input or Kohonen layer contains information on input values which are vector represented chemical structure. The structure of *s*-th compound represented by *m *structural descriptors or "variables" can be expressed as ***X***_*s *_= (*x*_*s*1_, *x*_*s*2_, ... *x*_*sm*_). The output layer is associated with the output values so called target ***T***_*s *_= (*t*_*s*1_, *t*_*s*2_, ... *t*_*sj*_...*t*_*sp*_) which is a *p*-component vector of zeros and ones. One dimensional target in our classification models expresses carcinogenicity class (P-positive = 1 and NP-not positive = 0). The neural network is trained to respond for each input structure representation ***X***_*s *_from the training set with the output vector ***Out***_*s *_identical to the target (class-vector) ***T***_*s*_.

The Kohonen input layer of the CP ANN consists of n_x _× n_y _neurons. After the learning, the objects are organized in such a way that similar objects are situated close to each other. It is to emphasize that only the input values participate in this phase of learning (unsupervised step). For this step no knowledge about the target vector is needed [64].

In the second step the positions of objects are projected to the output layer, where the weights are adjusted to output values (supervised step). The trained output layer consists of n_x _× n_y _output neurons arranged in squared neighborhood. After the training, each weight of the output neurons *out*_*j *_is a real number between 0.0 and 1.0. For the final prediction of classes the response surface values must be again transformed into discrete values, 0 and 1. The threshold value between 0.01 and 0.99 must be determined for each class.

More detailed description of CPANN can be found in the literature [65-67].

### Models validation

#### Parameters used for evaluation of classification model

A common way to evaluate the performance of classification model (or classifier) is to employ a confusion matrix (see Table 10) according to the method of Cooper et al. [68]. In the confusion matrix the four different possible outcomes of a single prediction for two-class problem are displayed. The rows represent the number of entries belonging to actual (observed) class, while the columns represent the entries belonging to predicted class. N_negative _and N_positive _are the number of negative (non-carcinogens) and positive compounds (carcinogens) in the dataset. TP denotes the number of true positives, and TN denotes the number of true negatives. FP (false positives) is the number of errors made by predicting a compound of being active (carcinogen) while it is not; FN (false negatives) is the number of incorrectly predicted negatives (non-carcinogens).

**Table 10:** Confusion matrix for two class classifier (P- positive and N- negative).

		Predicted		
		
		Non-carcinogens (Negative)	Carcinogens (Positive)	Total predicted
**Observed**	**Non-carcinogens** (Negative)	**T**N	**F**P	**N_negative _= T**N + **F**P
	
	**Carcinogens** (Positive)	**F**N	**T**P	**N_positive _= F**N + **T**P
	
	Total observed	**T**N + **F**N	**F**P + **T**P	**N_total _= N_negative _+ N_positive_**

*Definitions in Table 6:

TP-True positive;

TN-True negative;

FP-False positive;

FN-False negative.

N_negative _is the number of negative (non-carcinogens) in the dataset;

N_positive _is the number of positive compounds (carcinogens) in the dataset;

N_total _is total number of negative (non-carcinogens) and positive compounds (carcinogens) in the dataset;

Cooper statistics express the ability of classification model to detect known active compounds (sensitivity), non-active compounds (specificity), and all chemicals in general (accuracy). See equations 1-3.

The main classification parameter is the *accuracy (AC) *(or concordance). It is determined using the equation:(1)

AC is defined as the total number of non-carcinogens and carcinogens correctly predicted among the total number of compounds.

The others statistical parameters of interest are *sensitivity, specificity, positive predictivity, negative predictivity, false negative rate, false positive rate *and etc. *Sensitivity *is defined as the percentage of correctly classified carcinogens among the total number of carcinogens. It can be determined as the *true positive rate *and can be expressed as follows:(2)

*Specificity *shows the percentage of correctly classified non-carcinogens among the total number of non-carcinogens and relates to *true negative rate*. The following equation corresponds to specificity:(3)

Training and test sets were composed for evaluation of models.*Training set *represents class values for learning. *Test set *represents class values for evaluation. Hypothesis were used to establish classification in the test set, which is compared to known one.

#### Internal validation

For evaluation *goodness-of-fit *or robustness of model the internal performance of model based on the training set (644 compounds) was applied. Several diagnostic statistical tools were implemented for characterization the *goodness-of-prediction *or predictability of obtained models. Firstly, statistical performance of test set (161 compounds) was calculated. Secondly, internal cross-validation [69,70] (CV) "leave 20% out" test was done. It was performed on a training set of 644 compounds, so that the set was divided into five training sets, each containing 80% of compounds, and five test sets with 20% of compounds. The sets were selected randomly in a way that each compound was exactly one time a part of the test set and four times a part of the training set.

#### External validation

External validation is commonly used for the predictivity and reliability of QSAR model [71,72]. Therefore the predictive performance of QSAR models should be evaluated using a validation set of compounds that were not used to generate the model. The validation set of 738 compounds was provided by the CAESAR project partner *(Istituto di Ricerche Farmacologiche "Mario Negri" (IRFMN), Milano, Italy) *and implemented for validation of models. The preparation of external validation set was performed and described in section Materials.

In conclusion, it should be highlighted that the evaluation of the classification system was done using the so-called internal training set (644 compounds) and test set (161 compounds), cross validation 20% out test, and external validation test set (738 compounds). The external test set included chemicals that were not considered in the modeling.

## Competing interests

The authors declare that they have no competing interests.

## Authors' contributions

NF compiled the data set, generated the models for classification of carcinogenic compounds, performed data analysis, and drafted the manuscript. MV provided the analyses of resulting predictions applying Cooper statistics. MN was responsible for the research organization within the Slovenian research team and provided the software for neural networks calculations. AR provided external validation set of carcinogenic compounds and contributed to statistical analyses of resulting predictions. EB was the coordinator of the CAESAR project, thus setting up the public models for predictions of five different endpoints of toxicity, and contributed in drafting of manuscript. All authors have read and approved the final manuscript.

## Supplementary Material

Additional file 1 **Table 1SI**. The list of 805 chemicals from CPDBAS used for carcinogenicity modeling. Click here for file
