# Supplementary material for: New public QSAR model for carcinogenicity
Source: Chem Cent J. 2010 Jul 29;4(Suppl 1):S3. doi: 10.1186/1752-153X-4-S1-S3 (PMC2913330; doi:10.1186/1752-153X-4-S1-S3)
Supplement: Additional file 1 — Table 1SI. The list of 805 chemicals from CPDBAS used for carcinogenicity modeling. [file 1752-153X-4-S1-S3-S1.doc]

#

# New public QSAR model for carcinogenicity

Natalja Fjodorova^1^*, Marjan Vračko^1^, Marjana Novič^1^, Alessandra Roncaglioni^2^; Emilio Benfenati^2^

**Supporting Information**

**Table of contents:**

**Table 1SI.** The list of 805 chemicals from CPDBAS used for carcinogenicity modeling.

**Table 1SI.** The list of 805 chemicals from CPDBAS used for carcinogenicity modeling.

*Definition of the fields in Table 1SI.

**ID_v5**- Code of the chemical used in CAESAR project, (ID of database version 5);

**ID_CPDBAS original** corresponds to ID number taken from Distributed Structure-Searchable Toxicity (DSSTox) Public Database Network <http://www.epa.gov/ncct/dsstox/sdf_cpdbas.html>;

**ChemName**: Chemical name is taken from DSSTox and double checked from PubChem Compound (NCBI) <http://www.ncbi.nlm.nih.gov/sites/entrez?db=pccompound>;

**CASRN**: Registry Number of the Chemical Abstract Service is taken from DSSTox and double checked from PubChem Compound (NCBI) <http://www.ncbi.nlm.nih.gov/sites/entrez?db=pccompound>;

**TD50_Rat**- Carcinogenic potency in rat. TD_50_ is the rate *in mg/kg body wt/day* which, if administered chronically for the standard lifespan of the species, will halve the probability of remaining tumorless throughout that period. The TD_50_ value reported is the harmonic mean of the most potent TD_50_ values from each positive experiment in the species. All the values were derived from the Carcinogenic Potency DataBase, <http://potency.berkeley.edu/cpdb.html>.

**P-**positive or active or carcinogen

**NP-**not positive or inactive or non carcinogen

**Set**: Training and Test (prediction) set.

| **ID_v5** | **ID_CPDBAS original** | **Chemical Name** | **CASRN** | **TD50_Rat_mg** | **P(positive)/NP-(not-positive)** | **Set** |
| --- | --- | --- | --- | --- | --- | --- |
| 1 | 2 | Acetaldehyde | 75-07-0 | 153 | P | Training |
| 2 | 4 | Acetaldehyde oxime | 107-29-9 | NP | NP | Training |
| 3 | 5 | Acetamide | 60-35-5 | 180 | P | Training |
| 4 | 7 | Acetohexamide | 968-81-0 | NP | NP | Training |
| 5 | 8 | Acetone[4-(5-nitro-2-furyl)-2-thiazolyl] hydrazone | 18523-69-8 | 6.05 | P | Training |
| 6 | 9 | Acetonitrile | 75-05-8 | NP | NP | Training |
| 8 | 11 | 1'-Acetoxysafrole | 34627-78-6 | 25 | P | Training |
| 10 | 17 | 1-Acetylaminofluorene | 28314-03-6 | NP | NP | Training |
| 11 | 18 | 2-Acetylaminofluorene | 53-96-3 | 1.22 | P | Training |
| 12 | 19 | 4-Acetylaminofluorene | 28322-02-3 | NP | NP | Training |
| 13 | 20 | 4-Acetylaminophenylacetic acid | 18699-02-0 | NP | NP | Training |
| 15 | 23 | Acrolein | 107-02-8 | NP | NP | Training |
| 16 | 24 | Acrolein diethylacetal | 3054-95-3 | NP | NP | Training |
| 17 | 25 | Acrolein oxime | 5314-33-0 | NP | NP | Training |
| 18 | 26 | Acronycine | 7008-42-6 | 0.505 | P | Training |
| 19 | 27 | Acrylamide | 79-06-1 | 3.75 | P | Training |
| 20 | 28 | Acrylic acid | 79-10-7 | NP | NP | Training |
| 21 | 29 | Acrylonitrile | 107-13-1 | 16.9 | P | Training |
| 22 | 31 | Actinomycin D | 50-76-0 | 0.00111 | P | Training |
| 23 | 32 | Adipamide | 628-94-4 | NP | NP | Training |
| 24 | 33 | AF-2 | 3688-53-7 | 29.4 | P | Training |
| 25 | 34 | Aflatoxicol | 29611-03-8 | 0.00247 | P | Training |
| 27 | 38 | Alclofenac | 22131-79-9 | NP | NP | Training |
| 28 | 39 | Aldicarb | 116-06-3 | NP | NP | Training |
| 29 | 43 | Allantoin | 97-59-6 | NP | NP | Training |
| 30 | 44 | Allyl alcohol | 107-18-6 | NP | NP | Training |
| 31 | 46 | Allyl glycidyl ether | 106-92-3 | NP | NP | Training |
| 34 | 49 | 1-Allyl-1-nitrosourea | 760-56-5 | 0.341 | P | Training |
| 35 | 52 | 1-Amino-2,4-dibromoanthraquinone | 81-49-2 | 46 | P | Training |
| 36 | 53 | 3-Amino-4-ethoxyacetanilide | 17026-81-2 | NP | NP | Training |
| 37 | 57 | 1-Amino-2-methylanthraquinone | 82-28-0 | 59.2 | P | Training |
| 38 | 58 | 2-Amino-5-(5-nitro-2-furyl)-1,3,4-oxadiazole | 3775-55-1 | 3.67 | P | Training |
| 39 | 59 | 2-Amino-5-(5-nitro-2-furyl)-1,3,4-thiadiazole | 712-68-5 | 0.662 | P | Training |
| 40 | 60 | 2-Amino-4-(5-nitro-2-furyl)thiazole | 38514-71-5 | 5.85 | P | Training |
| 41 | 62 | 2-Amino-4-nitrophenol | 99-57-0 | 839 | P | Training |
| 43 | 64 | 4-Amino-2-nitrophenol | 119-34-6 | 309 | P | Training |
| 44 | 66 | 2-Amino-5-nitrothiazole | 121-66-4 | 44.6 | P | Training |
| 45 | 68 | 2-Aminoanthraquinone | 117-79-3 | 101 | P | Training |
| 46 | 69 | o-Aminoazotoluene | 97-56-3 | 4.04 | P | Training |
| 47 | 70 | 6-Aminocaproic acid | 60-32-2 | NP | NP | Training |
| 48 | 74 | 1-(Aminomethyl)cyclohexaneacetic acid | 60142-96-3 | 5850 | P | Training |
| 49 | 76 | 3-Aminotriazole | 61-82-5 | 9.94 | P | Training |
| 51 | 81 | Amobarbital | 57-43-2 | NP | NP | Training |
| 52 | 84 | 1-Amyl-1-nitrosourea | 10589-74-9 | 0.55 | P | Training |
| 54 | 89 | Anilazine | 101-05-3 | NP | NP | Training |
| 57 | 97 | Aramite | 140-57-8 | 96.7 | P | Training |
| 58 | 106 | L-Ascorbic acid | 50-81-7 | NP | NP | Training |
| 59 | 107 | Aspartame | 22839-47-0 | NP | NP | Training |
| 60 | 108 | Acetylsalicylic acid | 50-78-2 | NP | NP | Training |
| 62 | 112 | Atrazine | 1912-24-9 | 31.7 | P | Training |
| 63 | 113 | Atropine | 51-55-8 | NP | NP | Training |
| 64 | 117 | 6-Azacytidine | 3131-60-0 | NP | NP | Training |
| 65 | 118 | Azaserine | 115-02-6 | 0.793 | P | Training |
| 66 | 119 | Azathioprine | 446-86-6 | NP | NP | Training |
| 67 | 120 | Azelnidipine | 123524-52-7 | NP | NP | Training |
| 68 | 122 | Azinphosmethyl | 86-50-0 | NP | NP | Training |
| 70 | 124 | Azoxymethane | 25843-45-2 | 0.0466 | P | Training |
| 71 | 125 | 1-Azoxypropane | 17697-55-1 | 0.000241 | P | Training |
| 72 | 126 | 2-Azoxypropane | 17967-53-9 | 0.00268 | P | Training |
| 74 | 129 | Barbituric acid | 67-52-7 | NP | NP | Training |
| 75 | 132 | Bemitradine | 88133-11-3 | 548 | P | Training |
| 76 | 133 | Benzalazine | 64896-26-0 | NP | NP | Training |
| 77 | 134 | Benzaldehyde | 100-52-7 | NP | NP | Training |
| 78 | 135 | Benzene | 71-43-2 | 169 | P | Training |
| 79 | 137 | Benzidine | 92-87-5 | 1.73 | P | Training |
| 80 | 139 | Benzo(a)pyrene | 50-32-8 | 0.956 | P | Training |
| 81 | 141 | Benzofuran | 271-89-6 | 424 | P | Training |
| 82 | 142 | 1,3,5-Triazine-2,4-diamine, 6-phenyl- | 91-76-9 | NP | NP | Training |
| 84 | 144 | Benzoin | 119-53-9 | NP | NP | Training |
| 85 | 147 | 1,2,3-Benzotriazole | 95-14-7 | NP | NP | Training |
| 86 | 151 | Benzyl acetate | 140-11-4 | NP | NP | Training |
| 87 | 152 | Benzyl alcohol | 100-51-6 | NP | NP | Training |
| 88 | 153 | Benzyl chloride | 100-44-7 | NP | NP | Training |
| 89 | 154 | o-Benzyl-p-chlorophenol | 120-32-1 | NP | NP | Training |
| 90 | 155 | Benzyl isothiocyanate | 622-78-6 | NP | NP | Training |
| 91 | 156 | Benzyl thiocyanate | 3012-37-1 | NP | NP | Training |
| 92 | 158 | 3-Benzylsydnone-4-acetamide | 14504-15-5 | 424 | P | Training |
| 93 | 164 | 2,2-Bis(bromomethyl)-1,3-propanediol, technical grade | 3296-90-0 | 111 | P | Training |
| 94 | 167 | Bis(2-chloro-1-methylethyl)ether, technical grade | 108-60-1 | NP | NP | Training |
| 96 | 175 | 1,4-Bis[2-(3,5-dichloropyridyloxy)]benzene | 76150-91-9 | NP | NP | Training |
| 97 | 176 | 4-Bis(2-hydroxyethyl)amino-2-(5-nitro-2-thienyl)quinazoline | 33372-39-3 | 3.14 | P | Training |
| 98 | 177 | 4-Bis(2-hydroxyethyl)amino-2-(2-thienyl)quinazoline | 58139-47-2 | NP | NP | Training |
| 99 | 179 | Diisopropanolamine | 110-97-4 | NP | NP | Training |
| 100 | 182 | Bisphenol A | 80-05-7 | NP | NP | Training |
| 102 | 191 | HC blue 1 | 2784-94-3 | 702 | P | Training |
| 103 | 193 | HC blue 2 | 33229-34-4 | NP | NP | Training |
| 104 | 198 | Bromodichloromethane | 75-27-4 | 72.5 | P | Training |
| 106 | 202 | Budesonide | 51333-22-3 | 0.291 | P | Training |
| 107 | 203 | 1,3-Butadiene | 106-99-0 | 261 | P | Training |
| 108 | 204 | tert-Butyl alcohol | 75-65-0 | 64.6 | P | Training |
| 110 | 206 | n-Butyl chloride | 109-69-3 | NP | NP | Training |
| 112 | 211 | di-tert-Butyl-4-hydroxymethyl phenol | 88-26-6 | NP | NP | Training |
| 113 | 212 | Phenol, 2-(1,1-dimethylethyl)-4-methyl- | 2409-55-4 | NP | NP | Training |
| 114 | 213 | N-Butyl-N'-nitro-N-nitrosoguanidine | 13010-08-7 | NP | NP | Training |
| 116 | 216 | Butylated hydroxytoluene | 128-37-0 | NP | NP | Training |
| 117 | 221 | Phenol, 4-(1,1-dimethylethyl)- | 98-54-4 | NP | NP | Training |
| 118 | 222 | N-Butylurea | 592-31-4 | NP | NP | Training |
| 119 | 223 | beta-Butyrolactone | 3068-88-0 | 13.8 | P | Training |
| 120 | 224 | Gamma-butyrolactone | 96-48-0 | NP | NP | Training |
| 122 | 232 | Caffeine | 58-08-2 | NP | NP | Training |
| 123 | 239 | Candesartan cilexetil | 145040-37-5 | NP | NP | Training |
| 124 | 240 | Caprolactam | 105-60-2 | NP | NP | Training |
| 125 | 242 | Captafol | 2425-06-1 | 73.4 | P | Training |
| 127 | 250 | Carbon tetrachloride | 56-23-5 | 2.29 | P | Training |
| 128 | 251 | Carboxymethylnitrosourea | 60391-92-6 | 4.31 | P | Training |
| 129 | 252 | Carbromal | 77-65-6 | NP | NP | Training |
| 130 | 253 | beta-Carotene | 7235-40-7 | NP | NP | Training |
| 132 | 259 | Celiprolol | 56980-93-9 | NP | NP | Training |
| 133 | 262 | Chloramben | 133-90-4 | NP | NP | Training |
| 134 | 263 | Chlorambucil | 305-03-3 | 0.896 | P | Training |
| 135 | 265 | Chloramphenicol | 56-75-7 | NP | NP | Training |
| 137 | 268 | Chlorendic acid | 115-28-6 | 40.8 | P | Training |
| 140 | 277 | 2-Chloro-5-(3,5-dimethylpiperidinosulphonyl)benzoic acid | 37087-94-8 | 4.85 | P | Training |
| 142 | 280 | 2-Chloronitrobenzene | 88-73-3 | NP | NP | Training |
| 143 | 281 | 4-Chloronitrobenzene | 100-00-5 | NP | NP | Training |
| 144 | 282 | 4-Chloro-m-phenylenediamine | 5131-60-2 | 315 | P | Training |
| 145 | 283 | 4-Chloro-o-phenylenediamine | 95-83-0 | 214 | P | Training |
| 146 | 286 | 3-Chloro-p-toluidine | 95-74-9 | NP | NP | Training |
| 147 | 287 | 5-Chloro-o-toluidine | 95-79-4 | NP | NP | Training |
| 148 | 289 | 2-Chloro-1,1,1-trifluoroethane | 75-88-7 | 87.3 | P | Training |
| 149 | 290 | (4-Chloro-6-(2,3-xylidino)-2-pyrimidinylthio) acetic acid (WY-14643) | 50892-23-4 | 4.36 | P | Training |
| 150 | 291 | 4-Chloro-6-(2,3-xylidino)-2-pyrimidinylthio(N-beta-hydroxyethyl)acetamide | 65089-17-0 | 6.49 | P | Training |
| 151 | 293 | 2-Chloroacetophenone (CN) | 532-27-4 | NP | NP | Training |
| 152 | 294 | 4-(Chloroacetyl)acetanilide | 140-49-8 | NP | NP | Training |
| 153 | 295 | p-Chloroaniline | 106-47-8 | NP | NP | Training |
| 154 | 297 | o-Chlorobenzalmalononitrile (CS) | 2698-41-1 | NP | NP | Training |
| 155 | 298 | Chlorobenzene | 108-90-7 | 247 | P | Training |
| 156 | 299 | Chlorobenzilate | 510-15-6 | NP | NP | Training |
| 157 | 300 | Chlorodibromomethane | 124-48-1 | NP | NP | Training |
| 161 | 307 | Chloromethyl methyl ether | 107-30-2 | 5.5 | P | Training |
| 164 | 313 | 1-(4-Chlorophenyl)-1-phenyl-2-propynyl carbamate | 10473-70-8 | 8.78 | P | Training |
| 165 | 314 | p-Chlorophenyl-2,4,5-trichlorophenyl sulfide | 2227-13-6 | NP | NP | Training |
| 166 | 316 | Chloroprene | 126-99-8 | 125 | P | Training |
| 167 | 319 | Chlorothalonil | 1897-45-6 | 2270 | P | Training |
| 168 | 320 | Chlorozotocin | 54749-90-5 | 0.0375 | P | Training |
| 169 | 322 | Chlorpropamide | 94-20-2 | NP | NP | Training |
| 171 | 329 | Cimetidine | 51481-61-9 | NP | NP | Training |
| 172 | 331 | Ciprofibrate | 52214-84-3 | 2.18 | P | Training |
| 173 | 332 | 1,2,3-Propanetricarboxylic acid, 2-hydroxy- | 77-92-9 | NP | NP | Training |
| 175 | 335 | Clobuzarit | 22494-47-9 | NP | NP | Training |
| 176 | 336 | Clofibrate | 637-07-0 | 169 | P | Training |
| 177 | 341 | Codeine | 76-57-3 | NP | NP | Training |
| 178 | 342 | Colcemid | 477-30-5 | NP | NP | Training |
| 179 | 343 | Compound 50-892 3-Hydroxyproquazone | 65765-07-3 | NP | NP | Training |
| 180 | 347 | Coumaphos | 56-72-4 | NP | NP | Training |
| 181 | 349 | m-Cresidine | 102-50-1 | 470 | P | Training |
| 182 | 350 | p-Cresidine | 120-71-8 | 98 | P | Training |
| 183 | 351 | Crotonaldehyde | 123-73-9 | 4.2 | P | Training |
| 184 | 354 | Guanidine, cyano- | 157480-33-6 | NP | NP | Training |
| 185 | 357 | Cyclocytidine | 31698-14-3 | NP | NP | Training |
| 186 | 358 | beta-Cyclodextrin | 7585-39-9 | NP | NP | Training |
| 188 | 363 | Cyclopentanone oxime | 1192-28-5 | 40.9 | P | Training |
| 189 | 364 | Cyclophosphamide | 50-18-0 | 2.21 | P | Training |
| 191 | 369 | Dacarbazine | 4342-03-4 | 0.71 | P | Training |
| 192 | 371 | 4,4'-Sulfonyldianiline (Dapsone) | 80-08-0 | 22.4 | P | Training |
| 193 | 373 | Tetrachlorodiphenylethane | 72-54-8 | NP | NP | Training |
| 194 | 374 | p,p'-Dichlorodiphenyl dichloroethylene | 72-55-9 | NP | NP | Training |
| 196 | 376 | Decabromodiphenyl oxide | 1163-19-5 | 3340 | P | Training |
| 197 | 378 | Deflazacort | 14484-47-0 | NP | NP | Training |
| 198 | 379 | Dehydroepiandrosterone | 53-43-0 | 83.5 | P | Training |
| 200 | 381 | Deltamethrin | 52918-63-5 | NP | NP | Training |
| 202 | 384 | Dexamethazone | 50-02-2 | NP | NP | Training |
| 203 | 389 | N-1-Diacetamidofluorene | 63019-65-8 | 19 | P | Training |
| 204 | 392 | Diallyl phthalate | 131-17-9 | NP | NP | Training |
| 205 | 395 | Diallylnitrosamine | 16338-97-9 | 33.9 | P | Training |
| 207 | 402 | 2,4-Diaminotoluene (2,4-toluene diamine) | 95-80-7 | 2.47 | P | Training |
| 209 | 407 | Diazinon | 333-41-5 | NP | NP | Training |
| 210 | 410 | Dibenzo-p-dioxin | 262-12-4 | NP | NP | Training |
| 211 | 411 | 3-Dibenzofuranamine | 4106-66-5 | 2.48 | P | Training |
| 212 | 413 | 1,2-Dibromo-3-chloropropane | 96-12-8 | 0.259 | P | Training |
| 213 | 414 | Dibromodulcitol | 10318-26-0 | 8.37 | P | Training |
| 214 | 415 | 1,2-Dibromoethane | 106-93-4 | 1.52 | P | Training |
| 215 | 417 | 5,7-Dibromoquinoline | 34522-69-5 | NP | NP | Training |
| 216 | 418 | 1,3-Dibutyl-1-nitrosourea | 56654-52-5 | 4.28 | P | Training |
| 217 | 422 | Ethane, 1,1-dichloro-1-fluoro- | 1717-00-6 | 5260 | P | Training |
| 219 | 428 | Dichloroacetic acid | 79-43-6 | 161 | P | Training |
| 220 | 429 | Dichloroacetylene | 7572-29-4 | 3.58 | P | Training |
| 222 | 431 | 1,4-Dichlorobenzene (p-dichlorobenzene) | 106-46-7 | 644 | P | Training |
| 223 | 432 | 3,3'-Dichlorobenzidine | 91-94-1 | 28.1 | P | Training |
| 224 | 435 | 2,7-Dichlorodibenzo-p-dioxin | 33857-26-0 | NP | NP | Training |
| 225 | 436 | Methane, dichlorodifluoro- | 75-71-8 | NP | NP | Training |
| 227 | 438 | 1,2-Dichloroethane | 107-06-2 | 8.04 | P | Training |
| 228 | 439 | 2,4-Dichlorophenol | 120-83-2 | NP | NP | Training |
| 230 | 448 | 1,2-Dichloropropane (propylene dichloride) | 78-87-5 | NP | NP | Training |
| 231 | 449 | Dichlorvos | 62-73-7 | 4.16 | P | Training |
| 233 | 451 | N,N'-Dicyclohexylthiourea | 1212-29-9 | NP | NP | Training |
| 234 | 452 | Dicyclopentadiene dioxide | 81-21-0 | NP | NP | Training |
| 235 | 454 | Photodieldrin | 13366-73-9 | NP | NP | Training |
| 237 | 458 | Chlorpyrifos (Dursban) | 2921-88-2 | NP | NP | Training |
| 240 | 462 | Diethylene glycol | 111-46-6 | 1660 | P | Training |
| 241 | 463 | Diethylformamide | 617-84-5 | NP | NP | Training |
| 242 | 464 | Diethylmaleate | 141-05-9 | NP | NP | Training |
| 243 | 465 | Diethylstilbestrol | 56-53-1 | 0.223 | P | Training |
| 245 | 467 | 2-(Difluoromethyl)-dl-ornithine | 70052-12-9 | NP | NP | Training |
| 246 | 470 | Diglycidyl resorcinol ether, technical grade | 101-90-6 | 3.78 | P | Training |
| 249 | 473 | 3,6-Dihydro-2-nitroso-2H-1,2-oxazine | 3276-41-3 | 90.6 | P | Training |
| 250 | 474 | 3,4-Dihydrocoumarin | 119-84-6 | 2970 | P | Training |
| 251 | 475 | Dihydrosafrole | 94-58-6 | 143 | P | Training |
| 252 | 478 | Dimethadione | 695-53-4 | NP | NP | Training |
| 253 | 479 | Dimethoate | 60-51-5 | NP | NP | Training |
| 255 | 482 | 2,5-Dimethoxy-4'-aminostilbene | 5803-51-0 | 0.721 | P | Training |
| 256 | 484 | 3,3'-Dimethoxybenzidine-4,4'-diisocyanate | 91-93-0 | 1630 | P | Training |
| 257 | 486 | 5,7-Dimethoxycyclopentene[c]coumarin | 1146-71-0 | NP | NP | Training |
| 258 | 487 | 5,7-Dimethoxycyclopentenone[2,3-c]coumarin | 1150-37-4 | NP | NP | Training |
| 259 | 488 | 5,7-Dimethoxycyclopentenone[3,2-c] coumarin | 1150-42-1 | NP | NP | Training |
| 260 | 489 | 5,6-Dimethoxysterigmatocystin | 65176-75-2 | 0.364 | P | Training |
| 262 | 491 | 4-Dimethylaminoazobenzene | 60-11-7 | 3.31 | P | Training |
| 263 | 492 | N,N'-Dimethyl-N,N'-dinitrosophthalamide | 3851-16-9 | NP | NP | Training |
| 264 | 493 | Dimethyl hydrogen phosphite | 868-85-9 | 139 | P | Training |
| 267 | 497 | 1,2-Dimethyl-5-nitroimidazole | 551-92-8 | 17 | P | Training |
| 268 | 498 | Dimethyl terephthalate | 120-61-6 | NP | NP | Training |
| 269 | 499 | N,N-Dimethylacetamide | 127-19-5 | NP | NP | Training |
| 270 | 502 | trans-2-[(Dimethylamino)methylimino]-5-[2-(5-nitro-2-furyl)vinyl]-1,3,4-oxadiazole | 55738-54-0 | 22.4 | P | Training |
| 271 | 507 | N,N-Dimethylaniline | 121-69-7 | 125 | P | Training |
| 272 | 509 | 5,5-Dimethylbarbituric acid | 24448-94-0 | NP | NP | Training |
| 273 | 514 | 1-Dodecanamine, N,N-dimethyl-, N-oxide | 1643-20-5 | NP | NP | Training |
| 274 | 515 | Dimethylformamide | 68-12-2 | NP | NP | Training |
| 275 | 516 | Dimethyl hydrazine (DMH) | 57-14-7 | NP | NP | Training |
| 276 | 518 | 2-(2,2-Dimethylhydrazino)-4-(5-nitro-2-furyl)thiazole | 26049-69-4 | 0.41 | P | Training |
| 277 | 519 | Dimethylnitramine | 4164-28-7 | 0.547 | P | Training |
| 278 | 520 | Dimethylvinyl chloride (DMVC) | 513-37-1 | 31.8 | P | Training |
| 279 | 522 | 2,4-Dinitro-6-tert-butylphenylmethanesulfonate | 29110-68-7 | NP | NP | Training |
| 280 | 525 | Dinitrosohomopiperazine | 55557-00-1 | 0.0615 | P | Training |
| 281 | 528 | 2,6-Dinitrotoluene | 606-20-2 | 0.292 | P | Training |
| 282 | 533 | 1,4-Dioxane | 123-91-1 | 267 | P | Training |
| 283 | 534 | Dioxathion | 78-34-2 | NP | NP | Training |
| 286 | 541 | 5,5-Diphenylhydantoin (phenytoin) | 57-41-0 | NP | NP | Training |
| 287 | 545 | 2,5-Dithiobiurea | 142-46-1 | NP | NP | Training |
| 289 | 547 | 3-O-Dodecylcarbomethylascorbic acid | NoCAS PubMed | NP | NP | Training |
| 290 | 549 | dl-Dopa | 63-84-3 | NP | NP | Training |
| 291 | 551 | Doxefazepam | 40762-15-0 | NP | NP | Training |
| 292 | 557 | Ellagic acid | 476-66-4 | NP | NP | Training |
| 294 | 566 | Epichlorhydrin | 106-89-8 | 2.96 | P | Training |
| 296 | 572 | Estazolam | 29975-16-4 | NP | NP | Training |
| 297 | 574 | Estradiol mustard | 22966-79-6 | NP | NP | Training |
| 298 | 576 | Ethinyl estradiol | 57-63-6 | 0.2 | P | Training |
| 300 | 580 | Dulcin | 150-69-6 | 537 | P | Training |
| 301 | 582 | Ethoxyquin | 91-53-2 | NP | NP | Training |
| 302 | 583 | Ethyl acrylate | 140-88-5 | 119 | P | Training |
| 305 | 586 | Z-Ethyl-O,N,N-azoxymethane | 57497-29-7 | 0.0189 | P | Training |
| 306 | 588 | S-Ethyl-l-cysteine | 2629-59-6 | NP | NP | Training |
| 307 | 589 | Di(p-ethylphenyl)dichloroethane | 72-56-0 | NP | NP | Training |
| 308 | 591 | Ethyl-3-methyl-3-phenylglycidate | 77-83-8 | NP | NP | Training |
| 310 | 596 | Ethylbenzene | 100-41-4 | 4350 | P | Training |
| 311 | 600 | Ethylene oxide | 75-21-8 | 21.3 | P | Training |
| 312 | 601 | Ethylene thiourea (ETU) | 96-45-7 | 8.13 | P | Training |
| 313 | 604 | 4-Vinyl-1-cyclohexene diepoxide | 106-87-6 | NP | NP | Training |
| 314 | 605 | 2-Ethylhexanol | 104-76-7 | NP | NP | Training |
| 315 | 606 | Di(2-ethylhexyl)adipate | 103-23-1 | NP | NP | Training |
| 316 | 607 | Di(2-ethylhexyl) phthalate | 117-81-7 | 716 | P | Training |
| 317 | 609 | 1-Ethylnitroso-3-(2-hydroxyethyl)-urea | 96724-44-6 | 0.522 | P | Training |
| 318 | 610 | 1-Ethylnitroso-3-(2-oxopropyl)-urea | 110559-84-7 | 0.181 | P | Training |
| 319 | 611 | Ethylnitrosocyanamide | 38434-77-4 | 3.68 | P | Training |
| 320 | 612 | Ethylphenylacetylurea | 90-49-3 | NP | NP | Training |
| 321 | 614 | Ethynodiol diacetate | 297-76-7 | NP | NP | Training |
| 322 | 615 | Etodolac | 41340-25-4 | NP | NP | Training |
| 324 | 620 | Fenthion | 55-38-9 | NP | NP | Training |
| 325 | 621 | Fenvalerate | 51630-58-1 | NP | NP | Training |
| 326 | 627 | Fluconazole | 86386-73-4 | 80.7 | P | Training |
| 327 | 628 | Fluometuron | 2164-17-2 | NP | NP | Training |
| 328 | 629 | N-(2-Fluorenyl)-2,2,2-trifluoroacetamide | 363-17-7 | 1.62 | P | Training |
| 329 | 632 | N-4-(4'-Fluorobiphenyl)acetamide | 398-32-3 | 1.01 | P | Training |
| 330 | 633 | 2-Fluoroethyl-nitrosourea | 69112-98-7 | 0.125 | P | Training |
| 332 | 636 | Fluvastatin | 93957-54-1 | 125 | P | Training |
| 334 | 638 | Formic acid 2-[4-(2-furyl)-2-thiazolyl]hydrazide | 31873-81-1 | NP | NP | Training |
| 336 | 640 | Formic acid 2-[4-(5-nitro-2-furyl)-2-thiazolyl]hydrazide | 3570-75-0 | 5.06 | P | Training |
| 337 | 641 | 1-Formyl-3-thiosemicarbazide | 2302-84-3 | NP | NP | Training |
| 338 | 644 | Fumonisin b1 | 116355-83-0 | 1.16 | P | Training |
| 340 | 646 | Furan | 110-00-9 | 0.396 | P | Training |
| 343 | 649 | Fusarenon-X | 23255-69-8 | NP | NP | Training |
| 344 | 650 | Gallic acid | 149-91-7 | NP | NP | Training |
| 345 | 651 | Gemcadiol | 35449-36-6 | NP | NP | Training |
| 346 | 652 | Gemfibrozil | 25812-30-0 | NP | NP | Training |
| 347 | 657 | Glu-P-1 | 67730-11-4 | 4.69 | P | Training |
| 350 | 665 | Glycidaldehyde | 765-34-4 | NP | NP | Training |
| 351 | 666 | Glycidol | 556-52-5 | 4.28 | P | Training |
| 352 | 667 | Glycine | 56-40-6 | 25700 | P | Training |
| 353 | 669 | Glycyrrhetinic acid | 471-53-4 | NP | NP | Training |
| 354 | 678 | Hematoxylin | 517-28-2 | 1000 | P | Training |
| 355 | 679 | Heptachlor | 76-44-8 | NP | NP | Training |
| 356 | 680 | Heptamethyleneimine | 1121-92-2 | NP | NP | Training |
| 358 | 682 | Hexachlorobenzene | 118-74-1 | 3.86 | P | Training |
| 359 | 683 | Hexachloro-1,3-butadiene | 87-68-3 | 65.8 | P | Training |
| 360 | 684 | alpha-1,2,3,4,5,6-Hexachlorocyclohexane | 319-84-6 | 11.2 | P | Training |
| 361 | 688 | Hexachlorocyclopentadiene | 77-47-4 | NP | NP | Training |
| 362 | 689 | Hexachloroethane | 67-72-1 | 55.4 | P | Training |
| 363 | 690 | Hexachlorophene | 70-30-4 | NP | NP | Training |
| 364 | 692 | Urotropine | 100-97-0 | NP | NP | Training |
| 365 | 693 | Hexamethylmelamine | 531-18-0 | 10.2 | P | Training |
| 366 | 694 | Hexamethylphosphoramide | 680-31-9 | 0.0344 | P | Training |
| 367 | 696 | Hexanamide | 628-02-4 | NP | NP | Training |
| 368 | 697 | 1-O-Hexyl-2,3,5-trimethylhydroquinone | 148081-72-5 | NP | NP | Training |
| 369 | 698 | N-Hexylnitrosourea | 18774-85-1 | 0.513 | P | Training |
| 370 | 699 | 4-Hexylresorcinol | 136-77-6 | NP | NP | Training |
| 373 | 706 | 2-Hydrazino-4-(p-nitrophenyl) thiazole | 26049-70-7 | 3.21 | P | Training |
| 374 | 707 | 2-Hydrazino-4-phenylthiazole | 34176-52-8 | NP | NP | Training |
| 375 | 710 | Hydrazobenzene | 122-66-7 | 5.59 | P | Training |
| 376 | 712 | Ethane, 2,2-dichloro-1,1,1-trifluoro- | 306-83-2 | 2370 | P | Training |
| 377 | 713 | Hydrochlorothiazide | 58-93-5 | NP | NP | Training |
| 378 | 714 | Hydrocortisone | 50-23-7 | NP | NP | Training |
| 379 | 718 | 3-Hydroxy-4-acetylaminobiphenyl | 4463-22-3 | NP | NP | Training |
| 380 | 719 | N-Hydroxy-2-acetylaminofluorene | 53-95-2 | 0.988 | P | Training |
| 381 | 720 | 3-Hydroxy-4-aminobiphenyl | 4363-03-5 | NP | NP | Training |
| 382 | 722 | 1-Hydroxyanthraquinone | 129-43-1 | 93.5 | P | Training |
| 383 | 724 | 1-(2-Hydroxyethyl)-3-[(5-nitrofurfurylidene)amino]-2-imidazolidinone | 5036-03-3 | 16.7 | P | Training |
| 384 | 725 | 1-(2-Hydroxyethyl)-nitroso-3-ethylurea | 96724-45-7 | 0.562 | P | Training |
| 386 | 727 | 4-(2-Hydroxyethylamino)-2-(5-nitro-2-thienyl)quinazoline | 33389-36-5 | 1.87 | P | Training |
| 387 | 729 | 1-(3-Hydroxypropyl)-1-nitrosourea | 71752-70-0 | 0.978 | P | Training |
| 389 | 731 | 1'-Hydroxysafrole | 5208-87-7 | 18.4 | P | Training |
| 390 | 732 | Ibuprofen | 15687-27-1 | NP | NP | Training |
| 391 | 733 | ICRF-159 | 21416-87-5 | 10.7 | P | Training |
| 392 | 734 | 3-((Imino((2,2,2-trifluoroethyl)amino)methyl)amino)1H-pyrazole-1-pentamide | 84545-30-2 | 1170 | P | Training |
| 393 | 739 | Indolidan | 100643-96-7 | 2.01 | P | Training |
| 394 | 740 | Indomethacin | 53-86-1 | 1.15 | P | Training |
| 395 | 742 | Iodoacetamide | 144-48-9 | NP | NP | Training |
| 396 | 743 | Iodoform | 75-47-8 | NP | NP | Training |
| 398 | 747 | Isatidine | 15503-86-3 | 0.716 | P | Training |
| 400 | 750 | Isobutyl nitrite | 542-56-3 | 54.1 | P | Training |
| 401 | 751 | N-Isobutyl-N'-nitro-N-nitrosoguanidine | 5461-85-8 | NP | NP | Training |
| 402 | 754 | Isomazole | 86315-52-8 | 70.5 | P | Training |
| 403 | 755 | Isoniazid | 54-85-3 | 150 | P | Training |
| 404 | 759 | Isophorone | 78-59-1 | 1210 | P | Training |
| 405 | 760 | Isophosphamide | 3778-73-2 | 0.739 | P | Training |
| 406 | 762 | Isopropanol | 67-63-0 | NP | NP | Training |
| 407 | 764 | Isopropyl-N-(3-chlorophenyl) carbamate | 101-21-3 | NP | NP | Training |
| 408 | 768 | Kaempferol | 520-18-3 | NP | NP | Training |
| 409 | 770 | Chlordecone (kepone) | 143-50-0 | 2.96 | P | Training |
| 410 | 771 | Ketoprofen | 22071-15-4 | NP | NP | Training |
| 411 | 772 | Lasiocarpine | 303-34-4 | 0.389 | P | Training |
| 412 | 778 | D-Limonene | 5989-27-5 | 204 | P | Training |
| 413 | 779 | Lithocholic acid | 434-13-9 | NP | NP | Training |
| 414 | 782 | Lonidamine | 50264-69-2 | NP | NP | Training |
| 417 | 785 | Loxtidine | 76956-02-0 | 479 | P | Training |
| 418 | 790 | Malaoxon | 1634-78-2 | NP | NP | Training |
| 420 | 792 | Maleic hydrazide | 123-33-1 | NP | NP | Training |
| 421 | 797 | D-Mannitol | 69-65-8 | NP | NP | Training |
| 422 | 798 | Mannitol nitrogen mustard | 576-68-1 | NP | NP | Training |
| 423 | 801 | MeIQx | 77500-04-0 | 1.66 | P | Training |
| 424 | 802 | Melamine | 108-78-1 | 735 | P | Training |
| 426 | 804 | Melphalan | 148-82-3 | 0.0938 | P | Training |
| 427 | 805 | dl-Menthol | 89-78-1 | NP | NP | Training |
| 428 | 806 | MER-25 | 67-98-1 | NP | NP | Training |
| 429 | 807 | 2-Mercaptobenzothiazole | 149-30-4 | 344 | P | Training |
| 430 | 810 | 6-Mercaptopurine | 50-44-2 | NP | NP | Training |
| 431 | 815 | Metepa | 57-39-6 | 4.46 | P | Training |
| 433 | 817 | Methaphenilene | 493-78-7 | NP | NP | Training |
| 434 | 820 | Methimazole | 60-56-0 | 1.14 | P | Training |
| 435 | 821 | dl-Methionine | 59-51-8 | NP | NP | Training |
| 438 | 826 | 3-Methoxycatechol | 934-00-9 | 48.7 | P | Training |
| 440 | 828 | Hydroquinone monomethyl ether | 150-76-5 | 658 | P | Training |
| 441 | 830 | 8-Methoxypsoralen | 298-81-7 | 32.4 | P | Training |
| 442 | 832 | Methyl bromide | 74-83-9 | NP | NP | Training |
| 443 | 833 | Methyl-t-butyl ether | 1634-04-4 | 702 | P | Training |
| 444 | 834 | Methyl carbamate | 598-55-0 | 56.6 | P | Training |
| 445 | 835 | Methyl carbazate | 6294-89-9 | NP | NP | Training |
| 446 | 836 | Methyl clofenapate | 21340-68-1 | 4.78 | P | Training |
| 447 | 838 | 3'-Methyl-4-dimethylaminoazobenzene | 55-80-1 | 3.28 | P | Training |
| 448 | 839 | N-Methyl-N,4-dinitrosoaniline | 99-80-9 | 1.3 | P | Training |
| 449 | 842 | Methyl linoleate hydroperoxide | 27323-65-5 | NP | NP | Training |
| 451 | 844 | Methyl methacrylate | 80-62-6 | NP | NP | Training |
| 452 | 846 | 1-Methyl-3-nitro-1-nitroso-guanidine | 70-25-7 | 0.803 | P | Training |
| 453 | 847 | 2-Methyl-1-nitroanthraquinone | 129-15-7 | 84.8 | P | Training |
| 454 | 848 | 4-Methyl-1-[(5-nitrofurfurylidene)amino]-2-imidazolidinone | 21638-36-8 | 5.34 | P | Training |
| 455 | 850 | N-Methyl-N-nitrosobenzamide | 63412-06-6 | 3.23 | P | Training |
| 456 | 851 | N-(N-Methyl-N-nitrosocarbamoyl)-l-ornithine | 63642-17-1 | 0.787 | P | Training |
| 457 | 853 | R(-)-2-Methyl-N-nitrosopiperidine *(S(+)-2-Methyl-N-nitrosopiperidine)* | 14026-03-0 | 13.2 | P | Training |
| 458 | 855 | Methyl parathion | 298-00-0 | NP | NP | Training |
| 459 | 856 | N-Methyl-2-pyrrolidone | 872-50-4 | NP | NP | Training |
| 460 | 859 | alpha-Methylbenzyl alcohol | 98-85-1 | 458 | P | Training |
| 461 | 861 | p-Methylcatechol | 452-86-8 | 248 | P | Training |
| 462 | 862 | 3-Methylcholanthrene | 56-49-5 | 0.491 | P | Training |
| 463 | 865 | 4,4'-Methylenebis(2-chloroaniline) | 101-14-4 | 19.3 | P | Training |
| 464 | 867 | 4,4'-Methylene-bis(2-methylaniline) | 838-88-0 | 7.38 | P | Training |
| 467 | 870 | Phenol, 2,2'-methylenebis[6-(1,1-dimethylethyl)-4-methyl- | 119-47-1 | NP | NP | Training |
| 471 | 879 | Methylnitramine | 598-57-2 | 17.4 | P | Training |
| 472 | 880 | 4-(Methylnitrosamino)-1-(3-pyridyl)-1-butanol | 76014-81-8 | 0.103 | P | Training |
| 473 | 881 | 4-(N-Nitroso-N-methylamino)-1-(3-pyridyl)-1-butanone | 64091-91-4 | 0.0999 | P | Training |
| 474 | 884 | Methylnitrosocyanamide | 33868-17-6 | 0.48 | P | Training |
| 475 | 885 | N-Methylolacrylamide | 924-42-5 | NP | NP | Training |
| 476 | 887 | 6-Methylquinoline | 91-62-3 | NP | NP | Training |
| 477 | 888 | 8-Methylquinoline | 611-32-5 | NP | NP | Training |
| 478 | 891 | Metiapine | 5800-19-1 | NP | NP | Training |
| 480 | 893 | Mexacarbate | 315-18-4 | NP | NP | Training |
| 481 | 894 | Michler's ketone | 90-94-8 | 5.64 | P | Training |
| 483 | 896 | Mirex, photo- | 39801-14-4 | 1.46 | P | Training |
| 484 | 897 | Misoprostol | 59122-46-2 | NP | NP | Training |
| 485 | 898 | Mitomycin C | 50-07-7 | 0.00102 | P | Training |
| 486 | 901 | Monochloroacetic acid | 79-11-8 | NP | NP | Training |
| 489 | 910 | Myleran | 55-98-1 | NP | NP | Training |
| 490 | 911 | Nafenopin | 3771-19-5 | 11 | P | Training |
| 491 | 912 | Nalidixic acid | 389-08-2 | 201 | P | Training |
| 492 | 916 | 1,5-Naphthalenediamine | 2243-62-1 | 69.6 | P | Training |
| 493 | 921 | 2-Naphthylamine | 91-59-8 | 61.6 | P | Training |
| 494 | 923 | Nefiracetam | 77191-36-7 | NP | NP | Training |
| 495 | 930 | Nicotine | 54-11-5 | NP | NP | Training |
| 496 | 936 | Nithiazide | 139-94-6 | 131 | P | Training |
| 497 | 939 | Nitrilotriacetic acid (NTA) | 139-13-9 | 1770 | P | Training |
| 498 | 942 | 3-Nitro-p-acetophenetide | 1777-84-0 | NP | NP | Training |
| 499 | 943 | 5-Nitro-o-anisidine | 99-59-2 | 53.9 | P | Training |
| 500 | 944 | Nitrofurazone | 59-87-0 | 6.98 | P | Training |
| 501 | 945 | 5-Nitro-2-furamidoxime | 772-43-0 | NP | NP | Training |
| 502 | 946 | 5-Nitro-2-furanmethanediol diacetate | 92-55-7 | NP | NP | Training |
| 503 | 947 | 3-(5-Nitro-2-furyl)-imidazo(1,2-alpha) pyridine | 75198-31-1 | 13.6 | P | Training |
| 504 | 948 | 5-(5-Nitro-2-furyl)-1,3,4-oxadiazole-2-ol | 2122-86-3 | 8.61 | P | Training |
| 506 | 950 | N-[5-(5-Nitro-2-furyl)-1,3,4-thiadiazol-2-yl]acetamide | 2578-75-8 | 8.84 | P | Training |
| 508 | 952 | N-[4-(5-Nitro-2-furyl)-2-thiazolyl]acetamide | 531-82-8 | 17.8 | P | Training |
| 509 | 953 | N-(4-(5-Nitro-2-furyl)-2-thiazolyl)formamide | 24554-26-5 | 4.25 | P | Training |
| 511 | 955 | 3-Nitro-3-hexene | 4812-22-0 | 8.66 | P | Training |
| 513 | 958 | 4-Nitro-o-phenylenediamine | 99-56-9 | NP | NP | Training |
| 514 | 959 | 5-Nitro-o-toluidine | 99-55-8 | NP | NP | Training |
| 516 | 961 | p-Nitroaniline | 100-01-6 | NP | NP | Training |
| 517 | 962 | o-Nitroanisole | 91-23-6 | 15.6 | P | Training |
| 518 | 963 | 4-Nitroanthranilic acid | 619-17-0 | NP | NP | Training |
| 519 | 964 | Nitrobenzene | 98-95-3 | 25.5 | P | Training |
| 520 | 965 | 6-Nitrobenzimidazole | 94-52-0 | NP | NP | Training |
| 522 | 967 | 1-Nitrobutane | 627-05-4 | NP | NP | Training |
| 523 | 968 | 2-Nitrobutane | 600-24-8 | 286 | P | Training |
| 525 | 970 | Nitrofen | 1836-75-5 | 420 | P | Training |
| 526 | 971 | 2-Nitrofluorene | 607-57-8 | 0.285 | P | Training |
| 527 | 972 | Nitrofurantoin | 67-20-9 | 163 | P | Training |
| 528 | 973 | 1-[(5-Nitrofurfurylidene)amino]-2-imidazolidinone | 555-84-0 | 5.26 | P | Training |
| 530 | 977 | Nitromethane | 75-52-5 | 40.4 | P | Training |
| 531 | 978 | 1-Nitronaphthalene | 86-57-7 | NP | NP | Training |
| 532 | 979 | 3-Nitropentane | 551-88-2 | 23.7 | P | Training |
| 533 | 980 | 1-Nitropropane | 108-03-2 | NP | NP | Training |
| 535 | 982 | 3-Nitropropionic acid | 504-88-1 | NP | NP | Training |
| 536 | 983 | 1-Nitropyrene | 5522-43-0 | 3.33 | P | Training |
| 538 | 985 | 8-Nitroquinoline | 607-35-2 | 9.82 | P | Training |
| 539 | 986 | Nitroso-Baygon | 38777-13-8 | 0.364 | P | Training |
| 540 | 987 | N-Nitroso-bis-(4,4,4-trifluoro-N-butyl)amine | 83335-32-4 | 0.748 | P | Training |
| 541 | 988 | 1-Nitroso-5,6-dihydrothymine | 62641-67-2 | NP | NP | Training |
| 542 | 989 | 1-Nitroso-5,6-dihydrouracil | 16813-36-8 | 0.0983 | P | Training |
| 543 | 990 | N-Nitroso-2,3-dihydroxypropyl-2-hydroxypropylamine | 89911-79-5 | 0.0535 | P | Training |
| 544 | 991 | Nitroso-2,3-dihydroxypropyl-2-oxopropylamine | 92177-50-9 | 0.0352 | P | Training |
| 545 | 992 | N-Nitroso-2,3-dihydroxypropylethanolamine | 89911-78-4 | 5.98 | P | Training |
| 546 | 993 | 1-Nitroso-3,5-dimethyl-4-benzoylpiperazine | 61034-40-0 | 9.66 | P | Training |
| 549 | 996 | 1-Nitroso-1-(2-hydroxypropyl)-3-chloroethylurea | 96806-35-8 | 0.873 | P | Training |
| 551 | 998 | N-Nitroso-3-hydroxypyrrolidine | 56222-35-6 | 7.65 | P | Training |
| 553 | 1000 | N-Nitroso-N-methyl-N-dodecylamine | 55090-44-3 | 0.537 | P | Training |
| 554 | 1001 | N-Nitroso-N-methyl-4-fluoroaniline | 937-25-7 | 0.255 | P | Training |
| 555 | 1002 | N-Nitroso-N-methyl-4-nitroaniline | 943-41-9 | NP | NP | Training |
| 557 | 1004 | N-Nitroso-N-methyl-N-tetradecylamine | 75881-20-8 | 1.65 | P | Training |
| 558 | 1005 | N-Nitroso-N-methyldecylamine | 75881-22-0 | 1.26 | P | Training |
| 559 | 1006 | N-Nitroso-N-methylurea | 684-93-5 | 0.0927 | P | Training |
| 560 | 1007 | 3-Nitroso-2-oxazolidinone | 38347-74-9 | 0.385 | P | Training |
| 562 | 1009 | di(N-Nitroso)-perhydropyrimidine | 15973-99-6 | 0.166 | P | Training |
| 563 | 1010 | Nitroso-1,2,3,6-tetrahydropyridine | 55556-92-8 | 0.0601 | P | Training |
| 564 | 1011 | N-Nitroso(2,2,2-trifluoroethyl) ethylamine | 82018-90-4 | 2.52 | P | Training |
| 565 | 1013 | 1-Nitroso-3,4,5-trimethylpiperazine | 75881-18-4 | 0.151 | P | Training |
| 566 | 1014 | N-Nitrosoallyl-2,3-dihydroxypropylamine | 88208-16-6 | 0.825 | P | Training |
| 567 | 1015 | N-Nitrosoallyl-2-hydroxypropylamine | 91308-70-2 | 0.877 | P | Training |
| 569 | 1017 | N-Nitrosoallylethanolamine | 91308-69-9 | 0.491 | P | Training |
| 572 | 1020 | N-Nitrosobenzthiazuron | 51542-33-7 | 1.13 | P | Training |
| 573 | 1021 | N-Nitrosobis(2-hydroxypropyl)amine | 53609-64-6 | 0.846 | P | Training |
| 574 | 1022 | N-Nitrosobis(2-oxopropyl)amine | 60599-38-4 | 0.491 | P | Training |
| 577 | 1026 | Nitrosodibutylamine | 924-16-3 | 0.691 | P | Training |
| 578 | 1027 | N-Nitrosodiethanolamine | 1116-54-7 | 3.17 | P | Training |
| 579 | 1028 | N-Nitrosodiethylamine | 55-18-5 | 0.0265 | P | Training |
| 580 | 1029 | N-Nitrosodimethylamine | 62-75-9 | 0.0959 | P | Training |
| 581 | 1030 | N-Nitrosodiphenylamine | 86-30-6 | 167 | P | Training |
| 582 | 1031 | p-Nitrosodiphenylamine | 156-10-5 | 201 | P | Training |
| 583 | 1032 | N-Nitrosodipropylamine | 621-64-7 | 0.186 | P | Training |
| 584 | 1033 | N-Nitrosodithiazine | 114282-83-6 | NP | NP | Training |
| 585 | 1034 | Nitrosododecamethyleneimine | 40580-89-0 | 10.9 | P | Training |
| 586 | 1035 | N-Nitrosoephedrine | 17608-59-2 | 95.2 | P | Training |
| 587 | 1036 | Nitrosoethylmethylamine | 10595-95-6 | 0.0503 | P | Training |
| 588 | 1037 | Nitrosoethylurethane | 614-95-9 | 0.0904 | P | Training |
| 589 | 1038 | N-Nitrosoguvacoline | 55557-02-3 | NP | NP | Training |
| 590 | 1039 | Nitrosoheptamethyleneimine | 20917-49-1 | 0.0378 | P | Training |
| 592 | 1042 | Nitrosohydroxyproline | 30310-80-6 | NP | NP | Training |
| 593 | 1043 | Nitrosoiminodiacetic acid | 25081-31-6 | NP | NP | Training |
| 594 | 1044 | N-Nitrosomethyl-2,3-dihydroxypropylamine | 86451-37-8 | 0.646 | P | Training |
| 595 | 1045 | N-Nitrosomethyl-(2-hydroxyethyl) amine | 26921-68-6 | 1.29 | P | Training |
| 596 | 1046 | N-Nitrosomethyl-(3-hydroxypropyl) amine | 70415-59-7 | 1.66 | P | Training |
| 597 | 1048 | N-Nitrosomethyl(2-oxopropyl)amine | 55984-51-5 | 0.0172 | P | Training |
| 598 | 1050 | 2-Nitrosomethylaminopyridine | 16219-98-0 | 0.214 | P | Training |
| 599 | 1051 | 3-Nitrosomethylaminopyridine | 69658-91-9 | NP | NP | Training |
| 600 | 1052 | 4-Nitrosomethylaminopyridine | 16219-99-1 | NP | NP | Training |
| 601 | 1053 | Nitrosomethylaniline | 614-00-6 | 0.142 | P | Training |
| 602 | 1055 | Nitrosomethylundecylamine | 68107-26-6 | 2.37 | P | Training |
| 603 | 1056 | N-Nitrosomorpholine | 59-89-2 | 0.109 | P | Training |
| 604 | 1057 | N'-Nitrosonornicotine-1-N-oxide | 78246-24-9 | 0.876 | P | Training |
| 605 | 1058 | Nitrosopipecolic acid | 4515-18-8 | NP | NP | Training |
| 609 | 1062 | N-Nitrosopyrrolidine | 930-55-2 | 0.799 | P | Training |
| 610 | 1063 | N-Nitrosothialdine | 81795-07-5 | 0.483 | P | Training |
| 613 | 1070 | Norharman | 244-63-3 | NP | NP | Training |
| 614 | 1073 | Ochratoxin A | 303-47-9 | 0.103 | P | Training |
| 615 | 1074 | Octachlorostyrene | 29082-74-4 | NP | NP | Training |
| 616 | 1079 | Oltipraz | 64224-21-1 | NP | NP | Training |
| 618 | 1086 | Oxamyl | 23135-22-0 | NP | NP | Training |
| 619 | 1087 | Oxazepam | 604-75-1 | NP | NP | Training |
| 620 | 1088 | N-(9-Oxo-2-fluorenyl)acetamide | 3096-50-2 | 6.17 | P | Training |
| 621 | 1089 | Oxolinic acid | 14698-29-4 | 167 | P | Training |
| 622 | 1090 | 1-(2-Oxopropyl)nitroso-3-(2-chloroethyl)urea | 110559-85-8 | NP | NP | Training |
| 623 | 1091 | 2-Oxopropylnitrosourea | 89837-93-4 | NP | NP | Training |
| 624 | 1092 | 1'-Oxosafrole | 30418-53-2 | NP | NP | Training |
| 625 | 1094 | 4,4'-Oxydianiline | 101-80-4 | 9.51 | P | Training |
| 626 | 1095 | Morpholine, 4-[(4-morpholinylthio)thioxomethyl]- | 13752-51-7 | 90.8 | P | Training |
| 627 | 1100 | Parathion | 56-38-2 | NP | NP | Training |
| 628 | 1101 | Patulin | 149-29-1 | NP | NP | Training |
| 629 | 1103 | Pentachloroanisole | 1825-21-4 | 24.8 | P | Training |
| 630 | 1104 | Pentachloroethane | 76-01-7 | NP | NP | Training |
| 633 | 1116 | Phenacetin | 62-44-2 | 1250 | P | Training |
| 634 | 1117 | Phenazone | 60-80-0 | 1230 | P | Training |
| 635 | 1119 | Phenesterin | 3546-10-9 | 0.523 | P | Training |
| 636 | 1120 | Phenethyl isothiocyanate | 2257-09-2 | NP | NP | Training |
| 638 | 1124 | Phenol | 108-95-2 | NP | NP | Training |
| 639 | 1125 | Phenolphthalein | 77-09-8 | 902 | P | Training |
| 640 | 1128 | 1-Phenyl-3,3-dimethyltriazene | 7227-91-0 | 2.31 | P | Training |
| 642 | 1131 | N-Phenyl-2-naphthylamine | 135-88-6 | NP | NP | Training |
| 643 | 1133 | (*E*)-7-Phenyl-7-(3-pyridyl)-6-heptenoic acid | 89667-40-3 | NP | NP | Training |
| 644 | 1134 | 1-Phenyl-2-thiourea | 103-85-5 | NP | NP | Training |
| 645 | 1135 | C.I Solvent yellow 14 | 842-07-9 | 29.4 | P | Training |
| 646 | 1136 | Phenylbutazone | 50-33-9 | 1160 | P | Training |
| 647 | 1138 | 1,4-Benzenediamine | 106-50-3 | NP | NP | Training |
| 648 | 1143 | Phenylethyl-3-methylcaffeate | 71835-85-3 | NP | NP | Training |
| 649 | 1145 | Phenyl glycidyl ether | 122-60-1 | 44 | P | Training |
| 651 | 1158 | Phthalamide | 88-96-0 | NP | NP | Training |
| 652 | 1159 | Phthalic anhydride | 85-44-9 | NP | NP | Training |
| 654 | 1162 | Pilocarpine | 92-13-7 | NP | NP | Training |
| 655 | 1163 | Pimaricin | 7681-93-8 | NP | NP | Training |
| 656 | 1164 | Piperazine | 110-85-0 | NP | NP | Training |
| 657 | 1165 | Piperidine | 110-89-4 | NP | NP | Training |
| 659 | 1168 | Piperonyl sulfoxide | 120-62-7 | NP | NP | Training |
| 660 | 1170 | Piroxicam | 36322-90-4 | NP | NP | Training |
| 661 | 1171 | Pivalolactone | 1955-45-9 | 211 | P | Training |
| 662 | 1179 | Practolol | 6673-35-4 | NP | NP | Training |
| 663 | 1181 | Prazepam | 2955-38-6 | NP | NP | Training |
| 665 | 1183 | Prednimustine | 29069-24-7 | 19.2 | P | Training |
| 666 | 1184 | Prednisolone | 50-24-8 | 1.53 | P | Training |
| 667 | 1188 | Probenecid | 57-66-9 | NP | NP | Training |
| 668 | 1189 | Procarbazine | 671-16-9 | 4.01 | P | Training |
| 671 | 1201 | Propyl gallate | 121-79-9 | NP | NP | Training |
| 672 | 1203 | N-Propyl-N'-nitro-N-nitrosoguanidine | 13010-07-6 | 1.31 | P | Training |
| 673 | 1204 | N-Propyl-N-nitrosourea | 816-57-9 | 3.77 | P | Training |
| 674 | 1205 | Propylene | 115-07-1 | NP | NP | Training |
| 675 | 1206 | 1,2-Propylene glycol | 57-55-6 | NP | NP | Training |
| 676 | 1207 | 1,2-Propylene oxide | 75-56-9 | 74.4 | P | Training |
| 677 | 1209 | 6-Propyl-2-thiouracil | 51-52-5 | 13.7 | P | Training |
| 678 | 1211 | Proresid | 1508-45-8 | NP | NP | Training |
| 679 | 1212 | Protocatechuic acid | 99-50-3 | NP | NP | Training |
| 681 | 1215 | Pyrazinamide | 98-96-4 | NP | NP | Training |
| 685 | 1227 | C.I. Pigment red 23 | 6471-49-4 | NP | NP | Training |
| 686 | 1236 | HC red 3 | 2871-01-4 | NP | NP | Training |
| 688 | 1238 | Resorcinol | 108-46-3 | NP | NP | Training |
| 689 | 1239 | trans-Retinoic acid | 302-79-4 | NP | NP | Training |
| 690 | 1240 | Retinol acetate | 127-47-9 | 125 | P | Training |
| 691 | 1241 | All-trans-retinyl palmitate | 79-81-2 | NP | NP | Training |
| 692 | 1242 | Retrorsine | 480-54-6 | 862 | P | Training |
| 693 | 1244 | Rifampicin | 13292-46-1 | NP | NP | Training |
| 695 | 1248 | Rotenone | 83-79-4 | NP | NP | Training |
| 696 | 1251 | Saccharin | 81-07-2 | NP | NP | Training |
| 698 | 1255 | Salbutamol | 18559-94-9 | 40 | P | Training |
| 699 | 1256 | Salicylazosulfapyridine | 599-79-1 | 1590 | P | Training |
| 702 | 1267 | Sesamol | 533-31-3 | 1350 | P | Training |
| 703 | 1277 | Sorbic acid | 110-44-1 | NP | NP | Training |
| 704 | 1280 | Sterigmatocystin | 10048-13-2 | 0.152 | P | Training |
| 706 | 1282 | Streptozotocin | 18883-66-4 | 0.963 | P | Training |
| 707 | 1286 | Styrene oxide | 96-09-3 | 55.4 | P | Training |
| 708 | 1287 | Succinic anhydride | 108-30-5 | NP | NP | Training |
| 709 | 1289 | Sulfallate | 95-06-7 | 26.1 | P | Training |
| 710 | 1292 | Sulfisoxazole | 127-69-5 | NP | NP | Training |
| 711 | 1294 | 3-Sulfolene | 77-79-2 | NP | NP | Training |
| 712 | 1295 | 4,4'-Sulfonylbisacetanilide | 77-46-3 | 55.6 | P | Training |
| 713 | 1296 | Suxibuzone | 27470-51-5 | NP | NP | Training |
| 714 | 1297 | Symphytine | 22571-95-5 | 1.91 | P | Training |
| 715 | 1299 | Chlorotrianisene | 569-57-3 | NP | NP | Training |
| 716 | 1304 | L-Taurine | 107-35-7 | NP | NP | Training |
| 717 | 1305 | Tegafur | 37076-68-9 | NP | NP | Training |
| 719 | 1311 | Phenol, 2,4,6-tris(1,1-dimethylethyl)- | 732-26-3 | NP | NP | Training |
| 720 | 1313 | 2,3,5,6-Tetrachloro-4-nitroanisole | 2438-88-2 | NP | NP | Training |
| 721 | 1314 | 2,2',5,5'-Tetrachlorobenzidine | 15721-02-5 | NP | NP | Training |
| 722 | 1315 | 2,3,7,8-Tetrachlorodibenzo-p-dioxin | 1746-01-6 | 0.0000457 | P | Training |
| 723 | 1317 | 1,1,1,2-Tetrachloroethane | 630-20-6 | NP | NP | Training |
| 724 | 1318 | 1,1,2,2-Tetrachloroethane | 79-34-5 | NP | NP | Training |
| 725 | 1319 | Tetrachloroethylene | 127-18-4 | 101 | P | Training |
| 726 | 1322 | Tetraethylthiuram disulfide | 97-77-8 | NP | NP | Training |
| 727 | 1324 | Ethane, 1,1,1,2-tetrafluoro- | 811-97-2 | 29900 | P | Training |
| 728 | 1325 | Tetrafluoroethylene | 116-14-3 | 107 | P | Training |
| 729 | 1326 | Tetrahydro-2-nitroso-2H-1,2-oxazine | 40548-68-3 | 24.3 | P | Training |
| 731 | 1328 | Tetrahydrofuran | 109-99-9 | 407 | P | Training |
| 732 | 1329 | 3,4,5,6-Tetrahydrouridine | 18771-50-1 | NP | NP | Training |
| 733 | 1332 | Tetramethylthiouram disulfide | 137-26-8 | NP | NP | Training |
| 734 | 1334 | Tetranitromethane | 509-14-8 | 0.447 | P | Training |
| 735 | 1335 | Thenyldiamine | 91-79-2 | NP | NP | Training |
| 736 | 1336 | Theophylline | 58-55-9 | NP | NP | Training |
| 737 | 1337 | Thiabendazole | 148-79-8 | NP | NP | Training |
| 738 | 1338 | Thiamphenicol | 15318-45-3 | NP | NP | Training |
| 739 | 1339 | Tris(aziridinyl)-phosphine sulfide (thio-tepa) | 52-24-4 | 0.164 | P | Training |
| 740 | 1340 | Thioacetamide | 62-55-5 | 11.5 | P | Training |
| 741 | 1341 | 4,4-Thiobis(6-tert-butyl-m-cresol) | 96-69-5 | NP | NP | Training |
| 742 | 1344 | 4,4'-Thiodianiline | 139-65-1 | 3.71 | P | Training |
| 743 | 1345 | beta-Thioguanidine deoxyriboside | 789-61-7 | 2.1 | P | Training |
| 744 | 1346 | Thiosemicarbazide | 79-19-6 | NP | NP | Training |
| 745 | 1347 | Thiouracil | 141-90-2 | 11.9 | P | Training |
| 746 | 1348 | Thiourea | 62-56-6 | 98.5 | P | Training |
| 747 | 1355 | dl-alpha-Tocopherol | 10191-41-0 | NP | NP | Training |
| 748 | 1356 | dl-alpha-Tocopheryl acetate | 7695-91-2 | NP | NP | Training |
| 750 | 1359 | Tolbutamide | 64-77-7 | NP | NP | Training |
| 751 | 1362 | o-Toluenesulfonamide | 88-19-7 | 3960 | P | Training |
| 753 | 1370 | Trenimon | 68-76-8 | 0.00504 | P | Training |
| 754 | 1371 | Triamcinolone acetonide | 76-25-5 | 0.053 | P | Training |
| 755 | 1373 | Triamterene | 396-01-0 | NP | NP | Training |
| 756 | 1374 | Tribromomethane | 75-25-2 | 648 | P | Training |
| 758 | 1377 | 1,1,2-Trichloro-1,2,2-trifluoroethane, technical grade | 76-13-1 | NP | NP | Training |
| 760 | 1379 | 2,4,6-Trichloroaniline | 634-93-5 | NP | NP | Training |
| 761 | 1380 | 1,1,2-Trichloroethane | 79-00-5 | NP | NP | Training |
| 762 | 1381 | 1,1,1-Trichloroethane, technical grade | 71-55-6 | NP | NP | Training |
| 763 | 1382 | Trichloroethylene | 79-01-6 | NP | NP | Training |
| 764 | 1384 | Trichlorofluoromethane | 75-69-4 | NP | NP | Training |
| 765 | 1385 | N-(Trichloromethylthio)phthalimide | 133-07-3 | NP | NP | Training |
| 766 | 1386 | 2,4,6-Trichlorophenol | 88-06-2 | 405 | P | Training |
| 767 | 1388 | 2,4,5-Trichlorophenoxyacetic acid | 93-76-5 | NP | NP | Training |
| 769 | 1390 | 1,2,3-Trichloropropane | 96-18-4 | 1.35 | P | Training |
| 770 | 1392 | Triethanolamine | 102-71-6 | NP | NP | Training |
| 771 | 1393 | Triethylene glycol | 112-27-6 | NP | NP | Training |
| 772 | 1394 | 2,2,2-Trifluoro-N-[4-(5-nitro-2-furyl)-2-thiazolyl]acetamide | 42011-48-3 | 6.79 | P | Training |
| 773 | 1395 | Trifluralin, technical grade | 1582-09-8 | NP | NP | Training |
| 774 | 1396 | Trimethadione | 127-48-0 | NP | NP | Training |
| 775 | 1397 | (+-)-7-(3,5,6-trimethyl-1,4-benzoquinon-2-yl)-7-phenylheptanoic acid | 112665-43-7 | NP | NP | Training |
| 776 | 1398 | 2,4,5-Trimethylaniline | 137-17-7 | 33.6 | P | Training |
| 778 | 1404 | Trimethylthiourea | 2489-77-2 | 25.8 | P | Training |
| 779 | 1406 | 1,3,5-Trinitrobenzene | 99-35-4 | NP | NP | Training |
| 780 | 1407 | 1,2,3-Propanetriol, trinitrate | 55-63-0 | 183 | P | Training |
| 781 | 1413 | Tris(2,3-dibromopropyl) phosphate | 126-72-7 | 3.83 | P | Training |
| 782 | 1414 | Tris(2-ethylhexyl)phosphate | 78-42-2 | NP | NP | Training |
| 783 | 1415 | Triisopropanolamine | 122-20-3 | NP | NP | Training |
| 784 | 1418 | dl-Tryptophan | 54-12-6 | NP | NP | Training |
| 785 | 1419 | L-Tryptophan | 73-22-3 | NP | NP | Training |
| 786 | 1421 | Turmeric (>98% curcurmin) | 458-37-7 | NP | NP | Training |
| 787 | 1424 | Uracil | 66-22-8 | 671 | P | Training |
| 788 | 1425 | Urapidil | 34661-75-1 | NP | NP | Training |
| 789 | 1426 | Urea | 57-13-6 | NP | NP | Training |
| 790 | 1427 | Urethane | 51-79-6 | 41.3 | P | Training |
| 791 | 1430 | Vinblastine | 865-21-4 | NP | NP | Training |
| 792 | 1431 | Vinyl acetate | 108-05-4 | 341 | P | Training |
| 793 | 1432 | Vinyl bromide | 593-60-2 | 18.5 | P | Training |
| 795 | 1435 | Ethene, fluoro- | 75-02-5 | 20 | P | Training |
| 797 | 1440 | 2-Pyrrolidinone, 1-ethenyl- | 88-12-0 | 12 | P | Training |
| 798 | 1442 | Voglibose | 83480-29-9 | NP | NP | Training |
| 799 | 1450 | C.I. Disperse yellow 3 | 2832-40-8 | 380 | P | Training |
| 800 | 1451 | Diarylanilide yellow | 6358-85-6 | NP | NP | Training |
| 803 | 1454 | C.I Vat yellow 4 | 128-66-5 | NP | NP | Training |
| 805 | 1460 | Zearalenone | 17924-92-4 | NP | NP | Training |
| 7 | 10 | Acetoxime | 127-06-0 | 12.1 | P | Test |
| 9 | 16 | 4-Acetylaminobiphenyl | 4075-79-0 | 1.18 | P | Test |
| 14 | 21 | N-acetylcysteine | 616-91-1 | NP | NP | Test |
| 26 | 35 | Aflatoxin-B1 | 1162-65-8 | 0.0032 | P | Test |
| 32 | 47 | Allyl isothiocyanate | 57-06-7 | 96 | P | Test |
| 33 | 48 | Allyl isovalerate | 2835-39-4 | 123 | P | Test |
| 42 | 63 | 2-Amino-5-nitrophenol | 121-88-0 | 111 | P | Test |
| 50 | 77 | 11-Aminoundecanoic acid | 2432-99-7 | 1100 | P | Test |
| 53 | 87 | Benzene, 1-methoxy-4-(1E)-1-propenyl- | 4180-23-8 | NP | NP | Test |
| 55 | 90 | Aniline | 62-53-3 | NP | NP | Test |
| 56 | 94 | o-Anthranilic acid | 118-92-3 | NP | NP | Test |
| 61 | 110 | Astemizole | 68844-77-9 | NP | NP | Test |
| 69 | 123 | Azobenzene | 103-33-3 | 24.1 | P | Test |
| 73 | 127 | 3'-Azido-3'-deoxythymidine (AIDS) | 30516-87-1 | 11600 | P | Test |
| 83 | 143 | Benzoic acid | 65-85-0 | NP | NP | Test |
| 95 | 173 | Bis(chloromethyl) ether | 542-88-1 | 0.00357 | P | Test |
| 101 | 188 | C.I. Disperse blue 1 | 2475-45-8 | 156 | P | Test |
| 105 | 199 | Bromoethane (ethyl bromide) | 74-96-4 | 149 | P | Test |
| 109 | 205 | Butyl benzyl phthalate | 85-68-7 | 1040 | P | Test |
| 111 | 210 | n-Butyl-N-(4-hydroxybutyl)nitrosamine | 3817-11-6 | 0.457 | P | Test |
| 115 | 214 | N-n-Butyl-N-nitrosourea | 869-01-2 | 0.517 | P | Test |
| 121 | 231 | 3,4-Dihydroxycinnamic acid | 331-39-5 | 297 | P | Test |
| 126 | 247 | Carbaryl | 63-25-2 | 14.1 | P | Test |
| 131 | 257 | Catechol | 120-80-9 | 84.7 | P | Test |
| 136 | 267 | Chlordane (analytical grade) | 57-74-9 | NP | NP | Test |
| 138 | 275 | 4-Chloro-4'-aminodiphenylether | 101-79-1 | 37.6 | P | Test |
| 139 | 276 | 3-Chloro-4-(dichloromethyl)-5-hydroxy-2(5H)-furanone(MX) | 77439-76-0 | 0.583 | P | Test |
| 141 | 278 | Dinitrochlorobenzene | 97-00-7 | NP | NP | Test |
| 158 | 301 | Methane, chlorodifluoro- | 75-45-6 | NP | NP | Test |
| 159 | 302 | Chloroethane | 75-00-3 | NP | NP | Test |
| 160 | 305 | Chlorofluoromethane | 593-70-4 | 27.5 | P | Test |
| 162 | 311 | Monuron | 150-68-5 | 131 | P | Test |
| 163 | 312 | (+-)-4-(2-chlorophenyl)-2-[2-(4-isobutylphenyl)ethyl]-6,9-dimethyl-6H-thieno[3,2-f][1,2,4]triazolo[4,3-a][1,4]diazepine | 117279-73-9 | NP | NP | Test |
| 170 | 328 | Danthron | 117-10-2 | 245 | P | Test |
| 174 | 333 | Citrinin | 518-75-2 | 7.48 | P | Test |
| 187 | 359 | Cyclohexanone | 108-94-1 | NP | NP | Test |
| 190 | 365 | Cyclosporin A | 59865-13-3 | NP | NP | Test |
| 195 | 375 | Dichlorodiphenyltrichloroethane (DDT) | 50-29-3 | 84.7 | P | Test |
| 199 | 380 | Dehydroepiandrosterone acetate | 853-23-6 | 31.4 | P | Test |
| 201 | 383 | Deserpidine | 131-01-1 | NP | NP | Test |
| 206 | 396 | 4,6-Diamino-2-(5-nitro-2-furyl)-s-triazine | 720-69-4 | 1.71 | P | Test |
| 208 | 406 | Diazepam | 439-14-5 | NP | NP | Test |
| 218 | 427 | 2,6-Dichloro-p-phenylenediamine | 609-20-1 | NP | NP | Test |
| 221 | 430 | 1,2-Dichlorobenzene (o-dichlorobenzene) | 95-50-1 | NP | NP | Test |
| 226 | 437 | 1,1-Dichloroethane | 75-34-3 | NP | NP | Test |
| 229 | 442 | 2,4-Dichlorophenoxyacetic acid | 94-75-7 | NP | NP | Test |
| 232 | 450 | Dicofol | 115-32-2 | NP | NP | Test |
| 236 | 455 | 1,2,3,4-Diepoxybutane DL | 298-18-0 | NP | NP | Test |
| 238 | 459 | Diethylacetamide | 685-91-6 | 8.85 | P | Test |
| 239 | 460 | Diethylacetylurea | NOCAS | NP | NP | Test |
| 244 | 466 | N,N'-Diethylthiourea | 105-55-5 | 24 | P | Test |
| 247 | 471 | 5,6-Dihydro-5-azacytidine | 62488-57-7 | NP | NP | Test |
| 248 | 472 | 1,2-Dihydro-2-(5-nitro-2-thienyl) quinazolin-4(3H)-one | 33389-33-2 | 1.53 | P | Test |
| 254 | 480 | Dimethoxane | 828-00-2 | 716 | P | Test |
| 261 | 490 | O,O-Dimethyl S-2(acetylamino)ethyl dithiophosphate, technical grade | 13265-60-6 | NP | NP | Test |
| 265 | 495 | Dimethyl morpholinophosphoramidate | 597-25-1 | 614 | P | Test |
| 266 | 496 | 4,6-Dimethyl-2-(5-nitro-2-furyl) pyrimidine | 59-35-8 | 1.39 | P | Test |
| 284 | 536 | Dipentylnitrosamine | 13256-06-9 | 4.03 | P | Test |
| 285 | 538 | N,N'-Diphenyl-p-phenylenediamine | 74-31-7 | NP | NP | Test |
| 288 | 546 | Dithiooxamide | 79-40-3 | NP | NP | Test |
| 293 | 561 | Endrin | 72-20-8 | NP | NP | Test |
| 295 | 569 | 1,2-Epoxybutane | 106-88-7 | 220 | P | Test |
| 299 | 578 | Ethionine (DL-ethionine) | 13073-35-3 | 4.97 | P | Test |
| 303 | 584 | Ethanol | 64-17-5 | 9110 | P | Test |
| 304 | 585 | Z-Ethyl-O,N,N-azoxyethane | 16301-26-1 | 0.022 | P | Test |
| 309 | 595 | 3-O-Ethylascorbic acid | 86404-04-8 | NP | NP | Test |
| 323 | 617 | Eugenol | 97-53-0 | NP | NP | Test |
| 331 | 634 | 5-Fluorouracil | 51-21-8 | NP | NP | Test |
| 333 | 637 | Formaldehyde | 50-00-0 | 1.35 | P | Test |
| 335 | 639 | Formic acid 2-(4-methyl-2-thiazolyl)hydrazide | 32852-21-4 | 14.4 | P | Test |
| 339 | 645 | 2-Furaldehyde semicarbazone | 2411-74-7 | NP | NP | Test |
| 341 | 647 | Furfural | 98-01-1 | 683 | P | Test |
| 342 | 648 | Furosemide | 54-31-9 | NP | NP | Test |
| 348 | 658 | Glu-P-2 | 67730-10-3 | 42.3 | P | Test |
| 349 | 664 | 3-Chloro-1,2-propanediol | 96-24-2 | NP | NP | Test |
| 357 | 681 | Heptylamine | 111-68-2 | NP | NP | Test |
| 371 | 704 | 2-Hydrazino-4-(p-aminophenyl) thiazole | 26049-71-8 | 1.03 | P | Test |
| 372 | 705 | 2-Hydrazino-4-(5-nitro-2-furyl)thiazole | 26049-68-3 | 3.19 | P | Test |
| 385 | 726 | 1-(2-Hydroxyethyl)-1-nitrosourea | 13743-07-2 | 0.244 | P | Test |
| 388 | 730 | 8-Hydroxyquinoline | 148-24-3 | NP | NP | Test |
| 397 | 745 | IQ | 76180-96-6 | 0.812 | P | Test |
| 399 | 748 | Isobutene | 115-11-7 | 3550 | P | Test |
| 415 | 783 | Lornoxicam | 70374-39-9 | NP | NP | Test |
| 416 | 784 | Lovastatin | 75330-75-5 | NP | NP | Test |
| 419 | 791 | Malathion | 121-75-5 | NP | NP | Test |
| 425 | 803 | Meloxicam | 71125-38-7 | NP | NP | Test |
| 432 | 816 | Methafurylene | 531-06-6 | NP | NP | Test |
| 436 | 822 | Methotrexate | 59-05-2 | NP | NP | Test |
| 437 | 825 | 2-Methoxy-3-aminodibenzofuran | 5834-17-3 | 29 | P | Test |
| 439 | 827 | Methoxychlor | 72-43-5 | NP | NP | Test |
| 450 | 843 | Methyl linoleate, native | 112-63-0 | NP | NP | Test |
| 465 | 868 | Methylene chloride | 75-09-2 | 724 | P | Test |
| 466 | 869 | 4,4'-Methylenebis(N,N-dimethyl)benzenamine | 101-61-1 | 16.4 | P | Test |
| 468 | 872 | Methylguanidine | 471-29-4 | NP | NP | Test |
| 469 | 873 | 7-Methylguanine | 578-76-7 | NP | NP | Test |
| 470 | 876 | Methylhydroquinone | 95-71-6 | NP | NP | Test |
| 479 | 892 | Metronidazole | 443-48-1 | 542 | P | Test |
| 482 | 895 | Mirex | 2385-85-5 | 1.77 | P | Test |
| 487 | 902 | Monocrotaline | 315-22-0 | 0.94 | P | Test |
| 488 | 908 | 4-Morpholino-2-(5-nitro-2-thienyl)quinazoline | 58139-48-3 | 5.03 | P | Test |
| 505 | 949 | N-{[3-(5-Nitro-2-furyl)-1,2,4-oxadiazole-5-yl]-methyl}acetamide | 36133-88-7 | 59.6 | P | Test |
| 507 | 951 | 4-(5-Nitro-2-furyl)thiazole | 53757-28-1 | 7.68 | P | Test |
| 510 | 954 | N,N'-[6-(5-Nitro-2-furyl)-S-triazine-2,4-diyl]bisacetamide | 51325-35-0 | 14.1 | P | Test |
| 512 | 957 | 2-Nitro-p-phenylenediamine | 5307-14-2 | NP | NP | Test |
| 515 | 960 | 5-Nitroacenaphthene | 602-87-9 | 8.67 | P | Test |
| 521 | 966 | p-Nitrobenzoic acid | 62-23-7 | 287 | P | Test |
| 524 | 969 | Nitroethane | 79-24-3 | NP | NP | Test |
| 529 | 975 | Nitrogen mustard | 51-75-2 | 0.0114 | P | Test |
| 534 | 981 | 2-Nitropropane | 79-46-9 | NP | NP | Test |
| 537 | 984 | 6-Nitroquinoline | 613-50-3 | NP | NP | Test |
| 547 | 994 | 1-Nitroso-1-hydroxyethyl-3-chloroethylurea | 96806-34-7 | 0.356 | P | Test |
| 548 | 995 | N-Nitroso-2-hydroxymorpholine | 67587-52-4 | NP | NP | Test |
| 550 | 997 | N-Nitroso-(2-hydroxypropyl)-(2-hydroxyethyl)amine | 75896-33-2 | 1.02 | P | Test |
| 552 | 999 | N-Nitroso-N-isobutylurea | 760-60-1 | 4.73 | P | Test |
| 556 | 1003 | Nitroso-N-methyl-N-(2-phenyl)ethylamine | 13256-11-6 | 0.00998 | P | Test |
| 561 | 1008 | Nitroso-2-oxopropylethanolamine | 92177-49-6 | 1.8 | P | Test |
| 568 | 1016 | N-Nitrosoallyl-2-oxopropylamine | 91308-71-3 | 0.335 | P | Test |
| 570 | 1018 | Nitrosoamylurethane | 64005-62-5 | 1.01 | P | Test |
| 571 | 1019 | Nitrosoanabasine | 1133-64-8 | 11.9 | P | Test |
| 575 | 1023 | N-Nitrosobis(2,2,2-trifluoroethyl) amine | 625-89-8 | NP | NP | Test |
| 576 | 1025 | N-Nitrosocimetidine | 73785-40-7 | NP | NP | Test |
| 591 | 1041 | 1-Nitrosohydantoin | 42579-28-2 | 43.8 | P | Test |
| 606 | 1059 | N-Nitrosopiperazine | 5632-47-3 | 8.78 | P | Test |
| 607 | 1060 | N-Nitrosopiperidine | 100-75-4 | 1.43 | P | Test |
| 608 | 1061 | Nitrosoproline | 7519-36-0 | NP | NP | Test |
| 611 | 1064 | N-Nitrosothiomorpholine | 26541-51-5 | 5.39 | P | Test |
| 612 | 1065 | o-Nitrosotoluene | 611-23-4 | 50.7 | P | Test |
| 617 | 1080 | Omeprazole | 73590-58-6 | 119 | P | Test |
| 631 | 1111 | N-Pentyl-N'-nitro-N-nitrosoguanidine | 13010-10-1 | NP | NP | Test |
| 632 | 1115 | Petasitenine | 60102-37-6 | 0.922 | P | Test |
| 637 | 1122 | Phenobarbital | 50-06-6 | NP | NP | Test |
| 641 | 1130 | 1-Phenyl-3-methyl-5-pyrazolone | 89-25-8 | NP | NP | Test |
| 650 | 1151 | o-Phenylphenol | 90-43-7 | 232 | P | Test |
| 653 | 1160 | Picloram, technical grade | 1918-02-1 | NP | NP | Test |
| 658 | 1166 | Piperonyl butoxide | 51-03-6 | 633 | P | Test |
| 664 | 1182 | Praziquantel | 55268-74-1 | NP | NP | Test |
| 669 | 1195 | Propane sultone | 1120-71-4 | 3.84 | P | Test |
| 670 | 1197 | Propiolactone | 57-57-8 | 1.46 | P | Test |
| 680 | 1214 | Purpurin | 81-54-9 | 678 | P | Test |
| 682 | 1217 | Pyrimethamine | 58-14-0 | NP | NP | Test |
| 683 | 1222 | p-Benzoquinone dioxime | 105-11-3 | 106 | P | Test |
| 684 | 1226 | C.I. Pigment red 3 | 2425-85-6 | 1170 | P | Test |
| 687 | 1237 | Reserpine | 50-55-5 | 0.306 | P | Test |
| 694 | 1245 | Ripazepam | 26308-28-1 | NP | NP | Test |
| 697 | 1254 | Safrole | 94-59-7 | 441 | P | Test |
| 700 | 1260 | Secalciferol (24*R*,25-Dihydroxyvitamin D3) | 55721-11-4 | NP | NP | Test |
| 701 | 1266 | Senkirkine | 2318-18-5 | 1.7 | P | Test |
| 705 | 1281 | Stevioside | 57817-89-7 | NP | NP | Test |
| 718 | 1310 | Terbutaline | 23031-25-6 | 410 | P | Test |
| 730 | 1327 | 1-trans-delta-9-Tetrahydrocannabinol | 1972-08-3 | NP | NP | Test |
| 749 | 1358 | Tolazamide | 1156-19-0 | NP | NP | Test |
| 752 | 1366 | p-Tolylurea | 622-51-5 | NP | NP | Test |
| 757 | 1375 | Tricaprylin | 538-23-8 | 5490 | P | Test |
| 759 | 1378 | Trichloroacetic acid | 76-03-9 | NP | NP | Test |
| 768 | 1389 | Trichlorfon | 52-68-6 | NP | NP | Test |
| 777 | 1402 | Benzene, 1,2,4-trimethyl- | 95-63-6 | 4350 | P | Test |
| 794 | 1434 | Ethene, chloro- | 75-01-4 | 6.11 | P | Test |
| 796 | 1439 | Vinylidene fluoride | 75-38-7 | NP | NP | Test |
| 801 | 1452 | C.I. pigment yellow 16 | 5979-28-2 | NP | NP | Test |
| 802 | 1453 | Butanamide, 2,2'-[(3,3'-dichloro[1,1'-biphenyl]-4,4'-diyl)bis(azo)]bis[N-(4-chloro-2,5-dimethoxyphenyl)-3-oxo- | 5567-15-7 | NP | NP | Test |
| 804 | 1458 | HC yellow 4 | 59820-43-8 | NP | NP | Test |
